# Advances in spatial transcriptomics and its applications in cancer research

**DOI:** 10.1186/s12943-024-02040-9

**Published:** 2024-06-20

**Authors:** Yang Jin, Yuanli Zuo, Gang Li, Wenrong Liu, Yitong Pan, Ting Fan, Xin Fu, Xiaojun Yao, Yong Peng

**Affiliations:** 1 Laboratory of Molecular Oncology, Frontiers Science Center for Disease-related Molecular Network, State Key Laboratory of Biotherapy and Cancer Center, West China Hospital, Sichuan University, Chengdu, 610041 China; 2 Department of Thoracic Surgery, The Public Health Clinical Center of Chengdu, Chengdu, 610061 China; 3 Frontier Medical Center, Tianfu Jincheng Laboratory, Chengdu, 610212 China

**Keywords:** High-throughput sequencing, Spatial transcriptomics, Cancer

## Abstract

Malignant tumors have increasing morbidity and high mortality, and their occurrence and development is a complicate process. The development of sequencing technologies enabled us to gain a better understanding of the underlying genetic and molecular mechanisms in tumors. In recent years, the spatial transcriptomics sequencing technologies have been developed rapidly and allow the quantification and illustration of gene expression in the spatial context of tissues. Compared with the traditional transcriptomics technologies, spatial transcriptomics technologies not only detect gene expression levels in cells, but also inform the spatial location of genes within tissues, cell composition of biological tissues, and interaction between cells. Here we summarize the development of spatial transcriptomics technologies, spatial transcriptomics tools and its application in cancer research. We also discuss the limitations and challenges of current spatial transcriptomics approaches, as well as future development and prospects.

## Background

Cancer remains a significant global health challenge. Despite considerable progresses over the past decades, cancer treatment still faces numerous obstacles, such as drug resistance, tumor recurrence and metastasis. To better understand the initiation, progression, and treatment response of cancer, scientists continually develop new methods and technologies for cancer research. For example, by employing high-throughput transcriptomics sequencing techniques, known as RNA sequencing (RNA-seq), researchers have uncovered numerous cancer-specific changes of gene expression in different tumors. However, bulk RNA-seq can’t obtain information about the tumor microenvironment (TME) and cell heterogeneity [1, 2].

The subsequent emergence of single-cell RNA sequencing (scRNA-seq) ushered in a new era that enables the study of the transcriptomes at the resolution of individual cells, facilitating in-depth analyses of cellular heterogeneity and the identification of distinct cell types. However, performing single-cell sequencing requires cells to be dissociated from the tissue, causing a loss of information about the spatial location of cells. Since the spatial location of cells may truly reflect the interaction between cells and is closely related to physiological and pathological functions, a new technology linking gene expression with spatial location information was highly anticipated.

In this context, spatial transcriptomics sequencing technology emerged as an ideal approach to elucidate cancer cell heterogeneity and spatial distributions within tissues. This cutting-edge technology enables the integration of cellular transcriptomic information with spatial coordinates within tissues, providing insights into the interactions among different cell types and the overall tissue architecture. Through the utilization of spatial transcriptomics, a more comprehensive understanding of tumor initiation and progression mechanisms has been obtained, offering novel perspectives and opportunities for cancer diagnosis and precision treatment.

This review summarizes the principles and methods of spatial transcriptomics, the latest advances in spatial transcriptomic technology, and its applications in the field of cancer research. It also discusses how to employ spatial transcriptomics to investigate tumor microenvironments and tumor heterogeneity and facilitate anti-cancer drug development.

## Advances in spatial transcriptomics techniques

Spatial transcriptomics is a technology that integrates imaging, biomarkers analysis, sequencing, and bioinformatics to precisely locate gene expression within tissue slices. It reveals the spatial distribution of diverse cell types in tissues, investigates the interactions between cell populations, and constructs gene expression maps of distinct tissue regions. This innovative technology holds tremendous promise for unraveling the intricate mechanisms underlying various diseases, including cancer. Spatial transcriptomics techniques can be categorized into three primary approaches (as shown in Table 1): laser capture microdissection-based approaches, in situ imaging-based approaches, and spatial indexing-based approaches [3].

**Table 1:** List of representative spatial transcriptomics technologies

Theory	Year	Methods	Spatial resolution		throughput	Sample Type	Advantages	Disadvantages	Reference
Laser capture microdissection-based approaches	1996	LCM	Cellular		low	frozen section/FFPE	Cells of interest can be manually selected	The operation is complicated and difficult	PMID: 8875945
	2014	TIVA	Cellular		low	living cell	Can be applied to living cells	Not suitable for the analysis of large numbers of cells, the analysis results are limited	PMID: 24412976
	2014	Tomo-seq	Cellular		low	frozen section	3D contour profile can be constructed	It requires multiple identical biological samples and is difficult to apply to human samples	PMID: 25417113
	2016	LCM-seq	Cellular		low	frozen section	Fixed cells are directly lysed without RNA extraction, simplifying the experimental procedure, and obtaining full-length transcriptome data	Only 200 to 300 cells can be captured	PMID: 27387371
	2017	GEO-seq	Cellular		low	frozen section	It is more sensitive than LCM-seq and can capture a larger number of cells	GEO-seq requires high-quality tissue sections and is expensive	PMID: 28207000
	2017	NICHE-seq	Cellular		high	frozen section	It can identify rare niche specific immune subsets and transcriptome maps in target areas	It can only determine the spatial location of specific ecological niches, relies on genetically engineered model organisms, and cannot be applied to human samples	PMID: 29217582
	2018	proximiD	Cellular		low	frozen section	It can recognize the characteristics of interactions between cells	Manual microdissection is labor-intensive and the analysis process is complex	PMID: 29786092
In situ imaging-based approaches	2012	RNAscope	subcellsular		low	frozen section/FFPE	High specificity, can detect low abundance of RNA molecules	The number of channels is limited and the flexibility is poor	PMID: 22166544
	2014	seqFISH	subcellsular		low	frozen section	High multiplicity	Specialized equipment is required, and small FOV long imaging limits the field of view	PMID: 24681720
	2015	MERFISH	Cellular		middle	frozen section	High multiplicity	Specialized equipment is required, and small FOV long imaging limits the field of view	PMID: 25858977
	2018	osmFISH	subcellsular		middle	frozen section	Larger tissue area can be detected, semi-automated, short hybridization time, high signal-to-noise ratio	The preparation and handling of tissue samples are highly demanding	PMID: 30377364
	2019	seqFISH+	Cellular		middle	frozen section	More advanced probe design and imaging technology than seqFISH, with a high signal-to-noise ratio	Specialized equipment is required, and small FOV long imaging limits the field of view	PMID: 30911168
	2022	CosMx	subcellsular		middle	frozen section/FFPE	CosMx enables in situ imaging of 6000 + RNA and 64 + protein molecules in tissue slices at single-cell and subcellular resolutions	It has high requirements for equipment and experimental operation technology	PMID: 36203011
	2013	ISS	subcellsular		low	frozen section/FFPE	SNV can be detected at subcellular resolution	Low sensitivity	PMID: 23852452
	2014	FISSEQ	subcellsular		low	frozen section/FFPE	All types of RNA can be captured	Small FOV	PMID: 24578530
	2021	HybISS	subcellsular		low	frozen section/FFPE	Combine ISH and ISS	Low detection efficiency and throughput	PMID: 34331448
	2021	ExSeq	subcellsular		low	frozen section/FFPE	Nanoscale resolution	Small FOV	PMID: 33509999
	2018	STARmap	subcellsular		middle	free/frozen section	No reverse transcription step required, high sensitivity	Low detection efficiency	PMID: 29190363
Spatial indexing-based approaches	2016	ST	100 μm		high	frozen section	High accessibility and total mRNA in complete tissue sections can be detected	Low resolution and low capture efficiency	PMID: 27365449
	2019	Slide-seq	10 μm		high	frozen section	high resolution	Low sensitivity	PMID: 30923225
	2019	HDST	2 μm		high	frozen section	high resolution	Sparse tissues require binning due to low detection sensitivity	PMID: 31501547
	2020	GeoMx	10 μm		high	frozen section/FFPE	Spatial multi-target techniques for tumor immunity and tumor microenvironment	Species involve only humans and mice, require manual selection of areas, and have low sensitivity	PMID: 32393914
	2020	DBiT-seq	2 μm		high	frozen section	Spatial multiomics profiles of mRNA and protein can be constructed simultaneously	Low sensitivity	PMID: 33188776
	2021	Slide-seq V2	10 μm		high	frozen section	Additional random barcodes have been introduced to improve data accuracy and coverage	Low sensitivity	PMID: 33288904
	2021	Seq-Scope		0.5 μm	high	frozen section	Enables visualization of transcriptome heterogeneity at cellular and subcellular levels in various tissues	Low sensitivity	PMID: 34115981
	2021	Stereo-seq		0.5 μm	high	frozen section	Has the largest capture area, with more sensitive and stronger mRNA capture capability	Low sensitivity	PMID: 35512705
	2021	sci-Space		Cellular	high	frozen section	It can reach the single-cell level	Only nuclear gene sequencing	PMID: 34210887

### Laser capture microdissection-based approaches

Laser capture microdissection (LCM) enables careful dissection of single cells from tissue sections with precision, providing cellular spatial information. The basic principle of this technique is to fix tissue slices on slides, isolate specific areas of interest from the tissue using LCM, and then capture the expression of genes in the isolated tissue via high-throughput sequencing. By analyzing the gene expression information from each separated cell or region, it is possible to reconstruct the spatial distribution and gene expression profiles of individual cells or regions within the tissue [4, 5]. The developed approaches combining LCM and RNA sequencing to obtain RNA profile with cellular spatial information include LCM-seq, TIVA, Tomo-seq, GEO-seq, NICHE seq, and proximiD (Table 1).

**Fig. 1:**
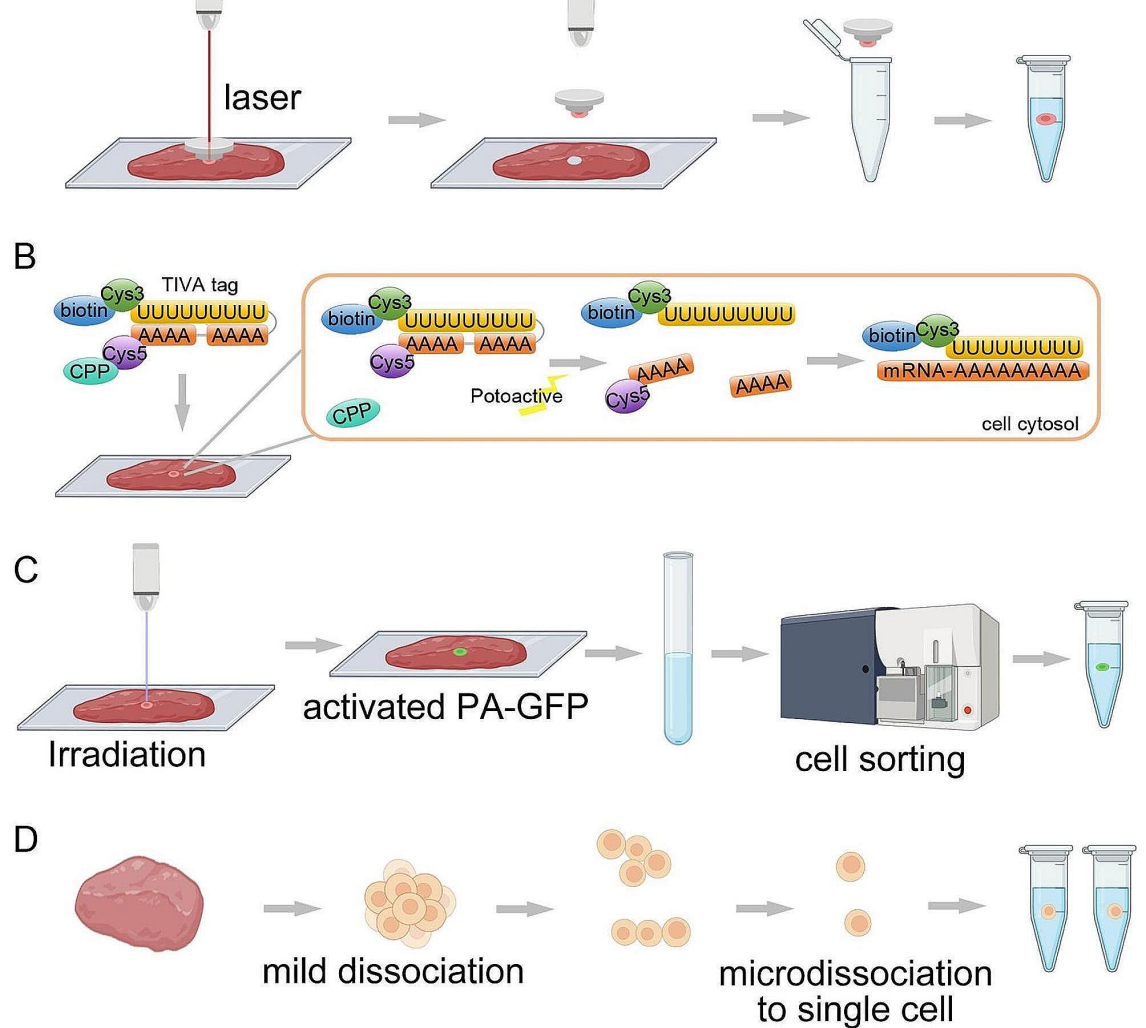
Spatial transcriptomics techniques based on laser capture microdissection. (**A**) In LCM, a laser cuts away cells of interest in tissue, then the target cells are transferred to the tube for library preparation. (**B**) In TIVA, A light-activated TIVA tags are loaded into cells or tissue, then photoactivation makes the TIVA-tags photolyzed in the desired cell, and the exposed poly(U) can anneal to the poly-A tail of cellular mRNA. (**C**) In NICHE-seq, PA-GFP tagged cells can be activated by irradiation, then activated cells are sorted to perform MARS-seq. (**D**) In ProximID, specimens are first mildly dissociated, and then dissociated into interacting structures, after that the units are microdissociated into single cells and placed in separate tubes for library preparation

LCM-seq, proposed by Nichterwitz et al., combines LCM with scRNA-seq, enabling the sequencing of RNA from individual cells or tissue regions (Fig. 1A) [6]. LCM-seq is relatively straightforward to perform but has lower throughput. Building on this, in 2017, Chen and colleagues proposed geographical position sequencing (GEO-seq), which similarly combines LCM with scRNA-seq but can cut and capture a larger number of cells per point compared to LCM-seq [7]. However, GEO-seq requires high-quality tissue sections and incurs higher costs.

Transcriptome in vivo analysis (TIVA) is the first noninvasive approach for capturing mRNA from live single cells in their natural microenvironment (Fig. 1B), which can be achieved by introducing biotin tags and photoactivating them [8]. The TIVA method is not suitable for the analysis of a large number of cells, and the TIVA label can only penetrate living cells, limiting its application on clinical samples. NICHE-seq uses light-activated fluorescent markers, two-photon laser scanning microscopy, and flow cytometry-based fluorescence-activated cell sorting in combination with scRNA-seq to reconstruct spatial organization (Fig. 1C). It can identify rare niche-specific immune subsets and transcriptomics profiles in target area [9]. However it is not appliable to human samples, as it can only determine the spatial location of specific ecological niches and relies on genetically engineered model organisms. ProximiD dissociates tissue under mild conditions. The interacting cellular structures are manually dissociated into single cells and analyzed separately [10]. It can characterize cell-cell interactions, but manual microdissection is labor-intensive, and the analysis process is complex (Fig. 1D).

In summary, LCM-based techniques can quantify the transcriptomes at the cellular level, but they fail to achieve higher resolution and can only track regional location information. In addition, LCM is very time-consuming, making high-throughput profiling of intricate tissues using these techniques but without extensive time a major challenge.

### In situ imaging-based approaches

**Fig. 2:**
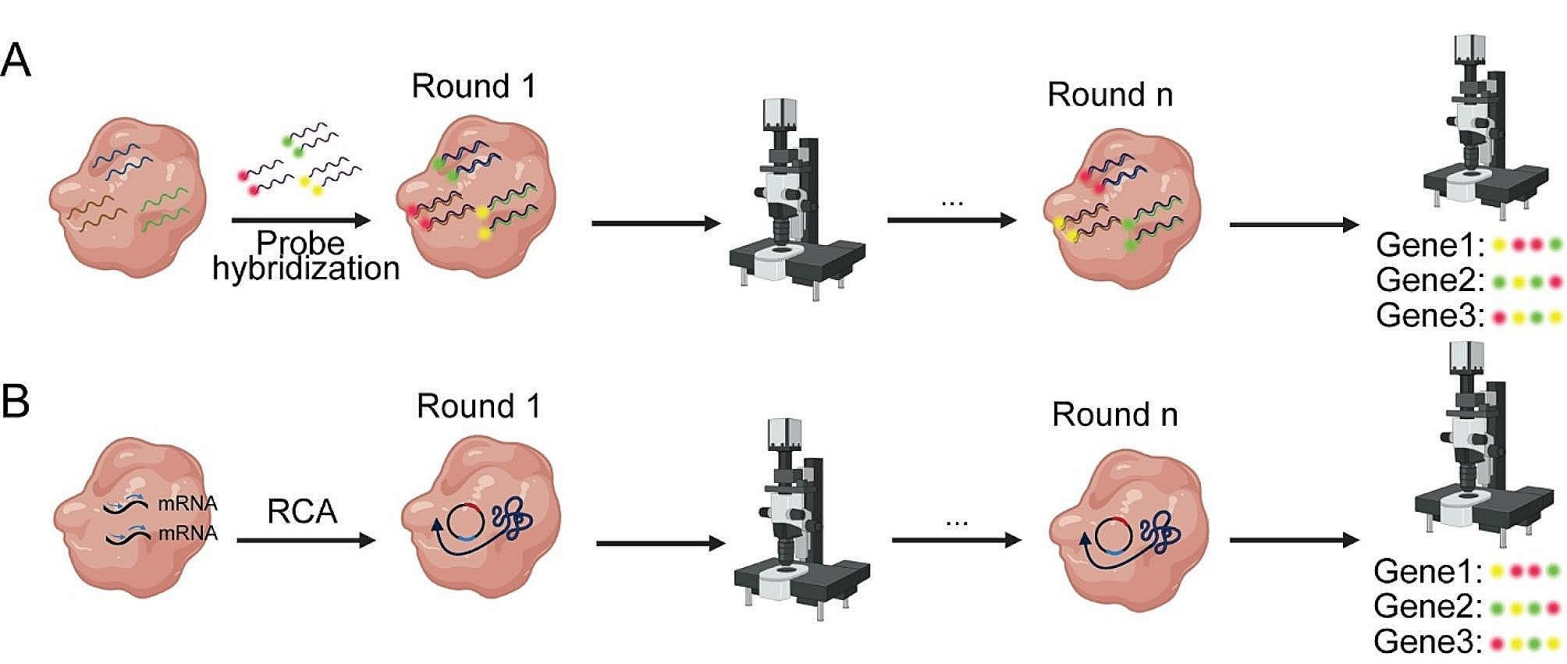
Spatial transcriptomics techniques based on in situ imaging. (A) Spatial transcriptomics technique based on in situ hybridization. (B) Spatial transcriptomics technology based on in situ sequencing

In situ imaging can also provide spatial information of cells. In situ hybridization (ISH) (Fig. 2A) and in situ sequencing (ISS) (Fig. 2B) are the two most commonly used methods.

ISH is based on the complementary nature of single-stranded nucleic acid molecules. It allows the detection of target RNA within cells or tissues through hybridization with corresponding oligonucleotide probes, which is then imaged and quantitatively located under a microscope [11]. Representative technologies include smFISH, RNA-scope, seqFISH, MERFISH, osmFISH, and seqFISH+ (Table 1).

The development of ISH can be traced back to the 1970s when the earliest methods involved the use of radiolabeled probes to analyze the localization of specific RNA within cells or tissues [11]. With the advancement of fluorescence labeling technology, the early radioactive labeling methods were gradually replaced [12]. Fluorescence in situ hybridization (FISH) is one of the most widely used ISH techniques. It utilizes fluorescently labeled oligonucleotide probes that hybridize with RNA within cells or tissues, followed by observation of the fluorescent signals under a fluorescence microscope to determine the spatial distribution of RNA. While FISH is relatively straightforward, Femino et al. combined FISH with digital imaging microscopy and developed single-molecule fluorescence in situ hybridization (smFISH), which enables quantitative RNA localization within single cells at subcellular resolution [13]. Subsequently, the RNAscope technique was developed, employing “Double-Z” probes designed to achieve signal amplification through a series of hybridization events with pre-amplifier molecules, amplifier molecules, and probes labeled with either fluorescent or enzyme chromogenic molecules [14]. This approach enables the detection of low-abundance RNA molecules. However, due to limitations posed by signal crosstalk, both smFISH and RNAscope typically allow detection of only a few RNA targets simultaneously.

Following smFISH and RNAscope, a series of methods have been developed, leading to an increase in both the throughput for cell number and the detection of target genes. One such advancement is the ouroboros single-molecule FISH (osmFISH), which allows the simultaneous detection of multiple genes from a large number of cells derived from tissue slices [15]. Sequential fluorescence in situ hybridization (seqFISH), building upon smFISH, involves multiple rounds of ISH with different barcodes. High-resolution imaging is used to detect the barcodes on each RNA molecule, determining both sequence information and spatial location [16]. SeqFISH can simultaneously detect hundreds of RNA molecules and is suitable for high-throughput detection in large samples. Subsequently, Eng et al. improved seqFISH and introduced seqFISH+ [17]. SeqFISH + uses a series of complementary DNA barcodes to label different RNA molecules, and these DNA barcodes can be amplified and sequenced to achieve super-resolution imaging of up to 10,000 genes. Concurrently, multiplexed error-robust fluorescence in situ hybridization (MERFISH) technology has developed rapidly [18]. It encodes each RNA molecule with unique fluorescent signals through multiplexed probe hybridization, followed by high-resolution fluorescence imaging to detect the spatial location and quantity of RNA molecules. This approach can simultaneously detect hundreds of RNA molecules and allows the detection of up to 40 different fluorescent probes in a single cell [19, 20].

While ISH technologies offer high-resolution imaging, they require multiple rounds of hybridization and imaging, increasing experimental time and sample handling complexity. Additionally, these techniques may suffer from cross-reactions, where the same probe hybridizes to multiple RNA molecules. They also face issues of probe hybridization noise, non-specific detection, and fluorescence drift, which can affect the accuracy of quantification and localization. Furthermore, these techniques are less effective in detecting novel or polymorphic transcripts since they rely on pre-designed and synthesized oligonucleotide probes. Detecting novelor polymorphic transcripts requires the redesign, validation, and optimization of new probes, adding complexity and increasing the time for experiments.

Compared to ISH, ISS can provide higher throughput. ISS technology captures transcripts within the native cellular environment at subcellular resolution, amplifying signals for sequencing using micro- or nano-sized DNA beads. Representative technologies include ISS, FISSEQ, HybISS, ExSeq and STARmap (Table 1).

In 2013, Ke et al. developed the lock-probe in situ sequencing (Lock-Seq) method [21]. In this technique, lock-probe sequences are hybridized on both sides of the target sequence to form a circular template, followed by continuous rolling-circle amplification (RCA) to amplify the signals for in situ sequencing. It revealed the correlation between sequencing information and its spatial location. However, Lock-Seq could only detect a limited number of RNA molecules, and lock-probes might introduce significant probe-specific biases, making it challenging to apply to the entire transcriptome. Subsequently, Lee et al. developed fluorescence in situ sequencing (FISSEQ) based on ISS. In this method, RNA molecules are first reverse-transcribed into complementary cDNA [22, 23]. The cDNA is then amplified and labeled with fluorescent markers, followed by multiple rounds of in situ sequencing. Optical imaging and computer analysis enable the determination of the spatial distribution of RNA molecules in tissues. FISSEQ has transformed targeted in situ sequencing into non-targeted sequencing, providing insights into gene expression, RNA splicing, and post-transcriptional modifications across the entire genome. However, FISSEQ has limitations in accurately detecting low-abundance transcripts and identifying variations and novel isoforms. Consequently, Gyllborg et al. proposed the hybridization-based in situ sequencing (HybISS) technology, which improved lock-probe design by replacing random primers with DNA oligonucleotide probes containing specific sequences, and replaced sequence-by-ligation (SBL) with sequence-by-hybridization (SBH) [24]. HybISS improved flexibility, signal-to-noise ratio, and specificity by using barcode probes, reduced false-positive signals, and achieved high spatial resolution and sensitivity at the single-cell level. Subsequently, Alon et al. combined tissue expansion with FISSEQ to develop the expansion sequencing (ExSeq) technology [25]. ExSeq utilizes expansion microscopy to physically enlarge biological samples, achieving high-precision in situ RNA-seq while maintaining the overall structural integrity of the samples. This method enables highly multiplexed RNA-seq from the nanoscale to the systems scale.

Additionally, in 2018, Wang et al. introduced the STARmap method, which employs barcode-lock probes for direct targeting of over a thousand genes [26]. STARmap’s advantage lies in its incorporation of a second primer. This allows the lock probe to bind to cDNA and initiate the rolling-circle amplification for in situ sequencing only when both primers align with the same mRNA molecule. This approach circumvents efficiency barriers associated with cDNA conversion and reduces noise by adding an additional hybridization step.

### Spatial indexing-based approaches

In recent years, with the advancement of high-throughput sequencing technologies, spatial transcriptomics based on in situ capture have gained widespread applications. The core principle of in situ capture technologies is to utilize DNA-barcoded primers with spatial positional information to capture and label the spatial locations of transcripts.

Ståhl et al. creatively applied ST technology to intact tissue sections by integrating high-throughput and unbiased total mRNA analysis with the morphological context of the tissue [27]. Their method covered over a thousand distinct capture regions, each with a unique spatial barcode. By reverse transcribing the mRNA in tissue sections and extracting cDNA-mRNA complexes, followed by library preparation and next-generation sequencing (NGS) readout, they successfully mapped gene expression data back to the tissue image using spatial barcodes and achieved spatial resolution for the entire transcriptome information. This innovative approach has been commercialized as Visium by 10x Genomics [28]. Visium combines microscopy and RNA sequencing to generate high-resolution mRNA expression data from stained tissue sections. It uses a chip containing thousands of RNA probes to capture RNA molecules in specific regions, each probe having a unique barcode for spatial RNA molecule localization. Visium does not require serial sectioning of tissues, avoiding the loss of RNA molecules. The technology offers the advantages of high throughput and high resolution.

**Fig. 3:**
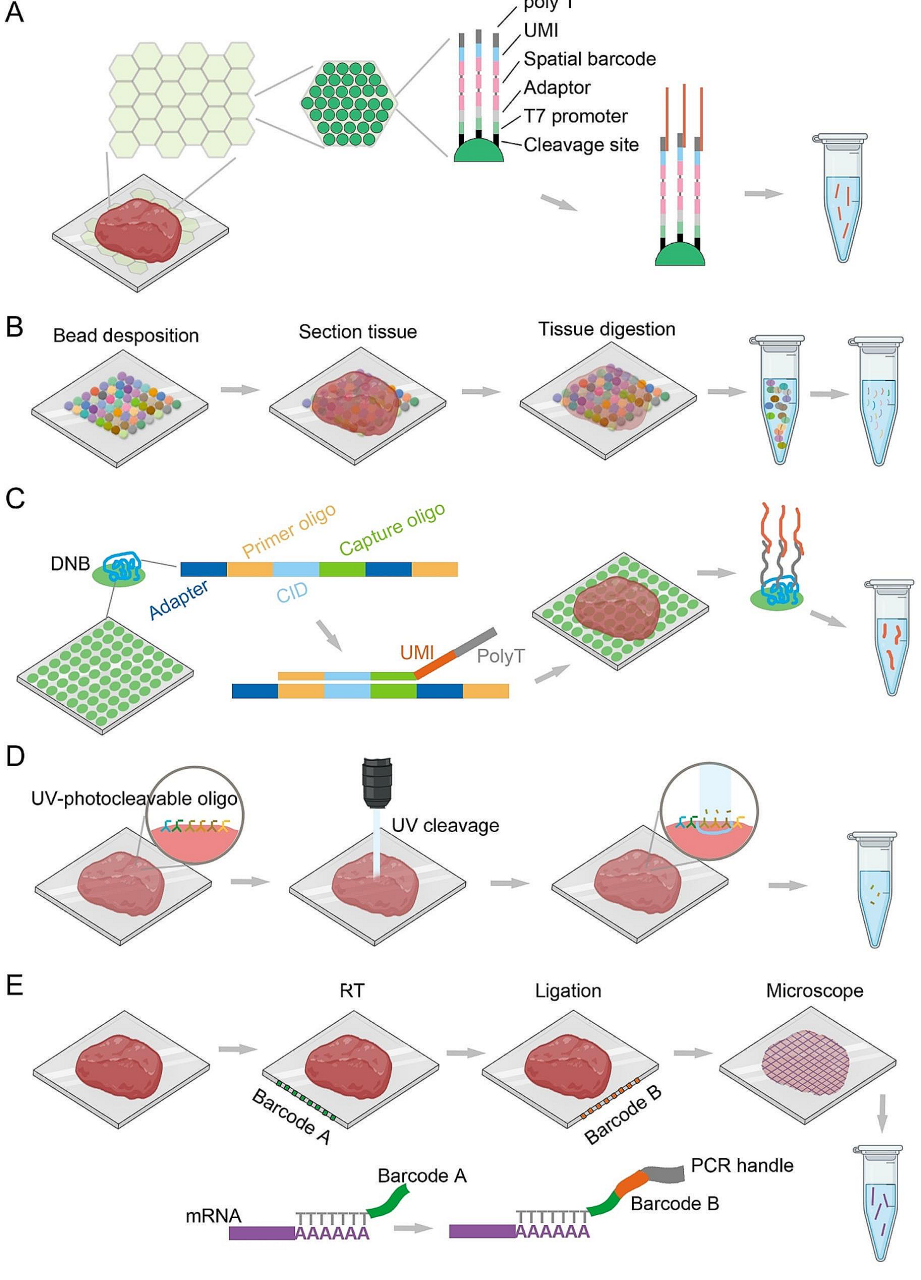
Spatial transcriptomics techniques based on spatial indexing. (**A**) In HDST, the barcoded poly(d)T oligonucleotides are deposited into 2 μm wells and their positions are decoded by a sequential hybridization strategy. After a tissue section is placed onto the slide, RNA is captured and collected for library preparation. (**B**) In Slide-seq, the slides are filled with DNA barcoded beads. The specimen is loaded on the slide and digested, then the barcoded RNAs are collected for library preparation. (**C**) In Stereo-seq, the DNA nanoball (DNB) containing random barcoded sequences are deposited onto the chip. Then the unique molecular indexes (UMI) and polyT oligonucleotides are ligated with the CID. After that, the tissue sections are load onto the chip surface, followed by fixation and permeabilization. Finally, the tissue polyA-tailed RNAs are captured and collected for library preparation. (**D**) In DSP, affinity reagents are covalently linked to UV-photocleavable oligonucleotides. The focused UV light liberates indexing oligonucleotides from any ROI. These oligonucleotides are then collected for library preparation. (**E**) In DBiT-seq, parallel microfluidic channels are used to deliver DNA barcodes to the surface of a tissue slide, yielding a 2-D mosaic of tissue pixels. ADTs recognize a panel of proteins of interest and co-mapping mRNAs

In 2019, Vickovic et al. introduced high-definition spatial transcriptomics (HDST) (Fig. 3A) [29]. In HDST, a tissue section is divided into hundreds of non-overlapping regions, incorporating magnetic beads with specific barcodes and multiple unique molecular indexes (UMIs) into each region for high-throughput RNA sequencing and spatial localization. HDST does not require specialized equipment or chemicals, making it suitable for standard histology laboratories. Although the 2 μm spot diameter of HDST approaches single-cell resolution, the captured transcript count is low, necessitating the analysis of spot grouping. In parallel, Slide-seq was developed, which is also based on histological images and RNA sequencing (Fig. 3B) [30]. In this method, a tissue sample is divided into hundreds of small regions, each containing specific RNA capture probes. When the probes bind to RNA in the tissue, a DNA barcode with a specific sequence is released. These barcodes can be associated with spatial information on the tissue section, allowing the spatial location of each RNA molecule to be determinated. Slide-seq offers high throughput and high resolution, capable of detecting transcriptomics information at the near single-cell level. However, it requires specific probes and chemical reagents, and its experimental operation can be relatively complex. Slide-seq V2 further improved this technology by introducing additional random barcodes to enhance data accuracy and coverage [31].

Furthermore, in 2021, Cho et al. achieved submicron-level spatial transcriptomics with Seq-Scope [32]. Seq-Scope uses a PCR-based in situ capture method to capture mRNA from individual cells and tissues at pre-defined spatial locations. These RNA molecules are then subjected to high-throughput sequencing, followed by analysis to determine their spatial location and expression levels within the tissue. Seq-Scope is fast, direct, precise, and easy to implement. It can analyze large numbers of samples in a matter of hours and can capture the expression of any gene within cells and tissues.

In 2021, sci-Space was developed. It works by spatially labeling nuclei with hashing oligonucleotides, transferring unique combinations of these oligos to tissue slices, and capturing spatial information during scRNA-seq [33]. sci-Space retains single-cell resolution while addressing spatial heterogeneity at larger scales. It can capture cell type-specific spatial patterns of gene regulation and estimate the contribution of each cell type to the expression of morphogens and signaling molecules within and across anatomical regions. This provides a valuable advantage for constructing spatially resolved single-cell atlases of mammalian development. In the same year, a research team at BGI developed Stereo-seq using an improved DNA nanoball (DNB) sequencing method (Fig. 3C). Stereo-seq utilizes DNB-patterned arrays and in situ RNA capture to achieve a large field-of-view spatial transcriptomics with single-cell resolution and high sensitivity [34]. Compared to other methods, Stereo-seq offers higher resolution, smaller spot size, and a greater number of spots, enabling detailed mapping of gene expression patterns at the cellular level. This innovative approach provides a comprehensive view of gene expression dynamics during organogenesis, serving as a valuable tool for analyzing spatial cell-type heterogeneity and cell fate specification.

Spatial transcriptomics techniques are not limited to RNA analysis and can also be applied to protein analysis. In 2019, Nanostring introduced GeoMx digital spatial profiling (GeoMx DSP), allowing simultaneous analysis of proteins and RNAs, providing the potential to acquire multi-dimensional information within cells [35]. This multi-modal analysis aids in gaining deeper insights into complex interactions within tissues, offering new opportunities for precision medicine and drug development (Fig. 3D). Rong Fan’s group developed deterministic barcoding in tissue (DBiT-seq) in 2020 [36]. Built upon microfluidic technology, the method achieves in situ reverse transcription within cells, facilitating the acquisition of transcriptomic and proteomic information through NGS and DNA barcoding (Fig. 3E). Spatial information is simultaneously obtained. As a novel spatial omics technique, DBiT-seq requires simple equipment and is user-friendly, facilitating its implementation by researchers.

The continuous development of these technologies has propelled advancements in the field of spatial transcriptomics, providing powerful tools for scientists to explore the complexity of biology within tissues.

### Spatial transcriptomics technique selection

Spatial transcriptomics technologies offer new insights into the spatial heterogeneity of gene expression. Each platform has its strengths and weaknesses, and we have focused on comparing several representative spatial transcriptomics sequencing technologies, including MERSCOPE (based on MERFISH [18]), CosMx (based on ISH [37]), Xenium (based on FISSEQ [23]), Visium [27], GeoMx [35], and STomics (based on Stereo-seq [34]). These platforms differ in their technical foundations, application scenarios, and suitability.

MERSCOPE, CosMx, and Xenium are imaging-based platforms capable of targeting specific mRNA lists, achieving single-cell and even subcellular resolution. ISH-based technologies like MERSCOPE and CosMx are more effective in detecting low-level mRNA compared to Xenium, which can examine larger tissue areas and directly obtain precise spatial information. This high spatial resolution makes them particularly suitable for detailed analysis of subcellular localization of specific genes and understanding intracellular and intercellular interactions with high precision. However, these technologies require longer imaging times and higher operational costs.

In contrast, Visium and STomics offer unbiased whole-genome coverage, suitable for large-scale mapping. Visium is widely used due to its relatively mature operation, but its spot diameter of 55 μm covers more than 25 cells, preventing single-cell resolution. STomics, based on Stereo-seq, provides comparable resolution (< 1 μm) to imaging-based methods. However, cDNA derived from this technology must be sequenced on proprietary sequencers rather than standard Illumina sequencers. GeoMx is ideal for spatial analysis of clinical and routine pathology samples. Its advantage lies in selecting ROIs based on functional tissue units or areas, though the total number of ROIs is limited, lacks single-cell specificity, and there is potential selection bias when manually choosing ROIs.

Researchers can choose the most suitable platform based on specific research needs to maximize research benefits and data quality.

## Bioinformatics tools of spatial transcriptomics

In recent years, there has been a rapid development in spatial transcriptomics data analysis techniques. From data preprocessing to the identification of spatially variable genes, clustering analysis, and downstream functional analysis, these steps can now be accomplished through a plethora of computational strategies designed in recent years. Here, we delineate the common analysis pipeline of spatial transcriptomics and compile the commonly used tools for upstream and downstream analysis of spatial transcriptommics data (as shown in Table 2).

**Table 2:** List of representative spatial transcriptomics bioinformatics tools

Tool	function	language	Description	Website	Refs
space ranger	data pre-processing(10X Visium)	shell	Space Ranger is a set of analysis pipelines that process 10x Genomics Visium data with brightfield or fluorescence microscope images, allowing users to map the whole transcriptome in a variety of tissues.	https://www.10xgenomics.com/support/software/space-ranger/latest	[43]
SAW	data pre-processing(Stereo-seq)	shell	Workflow for analyzing Stereo-seq transcriptomics data	https://github.com/STOmics/SAW	[44]
starfish	data pre-processing(ISS/ISH)	python	It is a scalable pipelines for image-based transcriptomics, can be utilized to localize and quantify RNA transcripts in image data	https://spacetx-starfish.readthedocs.io/en/latest/index.html	[38, 39]
Seurat	Comprehensive tool	R	Seurat is a toolbox for scRNA-seq and ST data analysis in R	https://github.com/satijalab/seurat	[40, 41]
Scanpy	Comprehensive tool	Python	Scanpy is a toolbox for scRNA-seq and ST data analysis in python	https://scanpy.readthedocs.io/en/stable/	[42]
Giotto	Comprehensive tool	R/Python	Gitto provides tools for processing, analyzing, and visualizing spatial multiomics data at various scales and multiple resolutions	https://github.com/RubD/Giotto	[45]
STUtility	Comprehensive tool	R	A versatile toolkit for spatially resolved transcriptomic analysis and visualization	https://github.com/jbergenstrahle/STUtility	[46]
Squidpy	Comprehensive tool	Python	It brings together tools from omics and image analysis to enable scalable description of spatial molecular data, such as transcriptome or multivariate proteins	https://github.com/scverse/squidpy	[47]
BayesSpace	Dimensionality reduction and clustering	R; C++	It’s a fully Bayesian statistical method that uses the information from spatial neighborhoods for resolution enhancement of spatial transcriptomics data and for clustering analysis.	https://github.com/edward130603/BayesSpace	[52]
SC-MEB	Dimensionality reduction and clustering	R; C++	spatial clustering with hidden Markov random field using empirical Bayes	https://github.com/Shufeyangyi2015310117/SC.MEB	[53]
SpaGCN	Dimensionality reduction and clustering	Python	SpaGCN is a graph convolutional network to integrate gene expression and histology to identify spatial domains and spatially variable genes.	https://github.com/jianhuupenn/SpaGCN	[54]
DeepST	Spatial domain identification	Python	DeepST is a method based on deep learning to identify spatial domains and enhance gene expression data	https://github.com/JiangBioLab/DeepST	[57]
STAGATE	Spatial domain identification	Python	STAGATE utilizes GAEs and GNNs to integrate and propagate spatial information from spatial transcriptomics data, enabling the effective identification of both local and global spatial domains.	https://github.com/QIFEIDKN/STAGATE	[58]
SiGra	Spatial domain identification	Python	SiGra uses single-cell spatial maps and graph transformation models to identify spatial domains based on imaging data	https://github.com/QSong-github/SiGra	[59]
trendsceek	Find HVGs	R	Trendsceek is a method based on marked point processes that identifies genes with statistically significant spatial expression trends.	https://github.com/edsgard/trendsceek	[60]
SpatialDE	Find HVGs	Python	SpatialDE is a method to identify genes which significantly depend on spatial coordinates in non-linear and non-parametric ways	https://github.com/Teichlab/SpatialDE	[61]
SPARK	Find HVGs	R; C++	SPARK is an efficient method to identify genes with spatial expression pattern	https://github.com/xzhoulab/SPARK	[62]
SPOTlight	Deconvolutin	R	SPOTlight is a tool that provides an NMFREg-based model for deconvolution of mixed cells from a single-cell reference.	https://github.com/MarcElosua/SPOTlight	[64]
SpatialDWLS	Deconvolutin	R	spatialDWLS uses damped least squares to determine the cell type composition of each spatial location	https://github.com/RubD/Giotto	[65]
Cell2location	Deconvolutin	Python	Cell2location is a Bayesian model-based tool that can parse fine-grained cell types in spatial transcriptomics data and create comprehensive cell maps of different tissues.	https://github.com/BayraktarLab/cell2location	[66]
CellTrek	Deconvolutin	R	CellTrek employs co-embedding and metric learning methods combined with spatial transcriptomics and single-cell transcriptomics data, to infer the distribution and relative abundance of cell types within tissues	https://github.com/navinlabcode/CellTrek	[67]
RCTD	Deconvolutin	R	RCTD combines sparse matrix factorization and tensor decomposition techniques for identifying and quantifying cell types and their relative spatial positions within tissues	https://github.com/dmcable/spacexr	[68]
DSTG	Deconvolutin	Python	DSTG deconvolves spatial transcriptomics data via graph-based convolutional networks to accurately deconvolve the gene expression observed at each point and restore its cellular structure	https://github.com/Su-informatics-lab/DSTG	[69]
GraphST	Deconvolutin	Python	GraphST is a self-supervised contrast learning method that combines graph neural networks with self-supervised contrast learning to learn informative and discriminative spots representations by minimizing the embedding distance between spatially adjacent spots	https://github.com/JinmiaoChenLab/GraphST	[70]
Tangram	Deconvolutin	Python	Tangram utilizes graph convolutional neural networks and modal adversarial training methods for spatial localization and deconvolution of cell types	https://github.com/broadinstitute/Tangram	[71]
STRIDE	Deconvolutin	Python	STRIDE is a deep learning-based method that integrates spatial transcriptomics and single-cell transcriptomics data for cell type identification and deconvolution of spatial distribution	https://github.com/wanglabtongji/STRIDE	[72]
stLearn	Spatial trajectory inference	Python	stLearn is a method that investigate complex biological processes within an undissociated tissue. It enables cell type identification, spatial trajectory reconstruction, and the study of cell-cell interactions in an undissociated tissue sample	https://github.com/BiomedicalMachineLearning/stLearn	[55]
SPATA	Spatial trajectory inference	R	SPATA analyzes dynamic changes using monocle3 to infer transcriptional patterns controlled by spatial organization dynamics	https://themilolab.github.io/SPATA2/	[73]
GCNG	Cellular interaction	Python	graph convolutional networks for inferring gene interaction from spatial transcriptomics data	https://github.com/xiaoyeye/GCNG	[74]
SpaOTsc	Cellular interaction	Python	SpaOTsc reconstructs spatial cellular dynamics within tissues by establishing a graph between spatial transcriptomics and single-cell transcriptomics datasets	https://github.com/zcang/SpaOTsc	[75]
MISTy	Cellular interaction	R	MISTy simulates interactions between labeled genes in neighboring cells by combining views from different spatial contexts	https://github.com/saezlab/mistyR	[76]
spaCI	Cellular interaction	Python	spaCI is an adaptive graph model that leverages spatial locations and gene expression profiles to identify ligand-receptor interactions in imaging-based spatial transcriptomics data	https://github.com/QSong-github/spaCI	[77]
Beyondcell	Drug discovery	R	Beyondcell is a method that uses ST data to identify drug vulnerabilities by scoring characteristic genes	https://github.com/cnio-bu/beyondcell	[78]
SpaRx	Drug discovery	Python	SpaRx utilizes graph-based domain adaptation to uncover spatial cellular response diversity to drugs by leveraging pharmacogenomic profiles	https://github.com/QSong-github/SpaRx	[79]

### Data pre-processing

The initial task in spatial transcriptomics data analysis is to obtain a gene expression matrix and corresponding spatial coordinates from the raw spatial transcriptomics dataset. These preprocessing steps typically rely on different techniques or platforms.

For image-based spatial transcriptomics data, common image processing steps include graph correction, stitching, registration, and the assignment of spatial context. Starfish [38, 39] offers scalable pipelines for image-based transcriptomics analysis. It can be used to localize and quantify RNA transcripts in image data and generate data formats suitable for subsequent Seurat [40, 41] or scanpy [42] analysis.

Preprocessing of sequencing-based spatial transcriptomics data primarily involves tissue image processing, sequencing file alignment, and matching sequencing results with spatial locations to ultimately generate a spatial gene expression matrix. Currently, commercial platforms like 10X Visium utilize Loupe Browser for tissue image processing and Space Ranger to convert sequencing data into gene expression matrices [43]. Stereo-seq employs ImageStudio for image quality assessment and manual adjustment, along with SAW to obtain spatial expression matrices suitable for downstream analysis [44].

Finally, the gene expression matrix and localization index matrix obtained through preprocessing serve as the starting point for downstream analysis.

### Generalized toolkits

Spatial data undergoes a variety of downstream analyses, including data normalization and batch effect correction, dimensionality reduction clustering, identification of spatially variable genes, cell type identification and deconvolution, spatial trajectory inference, and cell-cell communication analysis. These different downstream analyses require different input formats. To simplify and standardize spatial analysis, comprehensive analysis tools have been developed to provide unified formats for these data, such as Seurat [40, 41], scanpy [42], Giotto [45], STUtility [46], and squidpy [47]. These tools offer standardized data storage formats for downstream analysis of spatial data, with the most commonly used Seurat and scanpy generating SeuratObject and anndata, respectively. These comprehensive tools provide built-in functions for filtering and data normalization of preprocessed matrices. For instance, Seurat includes the SCTransform [48] function for normalization of UMI count data across spots. Additionally, these tools offer some downstream analysis and visualization capabilities. Compared to the relatively basic general functionalities of Seurat and scanpy, Giotto, STUtility, and squidpy, as specialized tools for spatial transcriptomics analysis, provide more professional and in-depth extended analysis functions. These specific functionalities are described in the following sections.

### Dimensionality reduction and clustering

Dimensionality reduction and clustering is a critical step in spatial transcriptomics analysis, aiding in the identification of distinct spatial features and providing a foundation for subsequent discovery of spatially associated genes and cell type identification. Traditional methods widely used in scRNA-seq analysis, such as PCA (Principal Component Analysis) [49], t-SNE (t-distributed Stochastic Neighbor Embedding) [50], and UMAP (Uniform Manifold Approximation and Projection) [51], are prevalent in various software packages. In recent years, novel methods have emerged, including BayesSpace [52] based on fully Bayesian statistical models, SC-MEB [53] based on Hidden Markov Random Fields (HMRF), and SpaGCN [54] based on Graph Convolutional Networks (GCN). Additionally, stLearn [55] leverages deep learning to infer spatial clustering and sub-clustering by incorporating tissue image information. Chen et al. conducted a comprehensive evaluation of different clustering methods [56].

### Spatial domain identification

Identifying spatial domains, which involves determining spatial points with consistent gene expression and histology, is considered a critical step in spatial transcriptomics analysis. This approach enables the revelation of intrinsic interactions and characteristics of the tissue microenvironment. DeepST employs Convolutional Neural Networks (CNN) to capture local spatial features, enabling the recognition of gene expression patterns at different spatial locations [57]. This model is particularly effective for analyzing local spatial features, though it is less adept at capturing long-range spatial dependencies and requires significant computational resources for training. In contrast, STAGATE represents the data as a graph structure, utilizing Graph Auto-Encoders (GAE) and Graph Neural Networks (GNN) to integrate and propagate spatial information, effectively capturing both global and local spatial features [58]. This approach is suited for modeling complex spatial relationships on a global scale, albeit with higher computational complexity. SiGra is a method developed to leverage imaging information for spatial transcriptomics data analysis. SiGra utilizes a single-cell spatial graph and a graph transformer model to identify spatial domains, effectively enhancing the understanding and analysis of spatial cellular ecosystems by revealing spatial patterns and boosting gene expression data [59]. The multimodal graph transformer framework of SiGra optimizes spatial transcriptomics data, unveiling spatial relationships between cells, and delving deeper into the intricate spatial architecture within tissues.

### Spatial characteristic gene identification

Identifying genes with spatially specific expression patterns can rapidly elucidate the characteristics of spatial organization. In Seurat, the FindSpatiallyVariableGenes function is employed to identify spatially variable genes (SVGs) through differential expression analysis. Other methods for identifying SVGs include trendsceek [60], SpatialDE [61], and SPARK [62]. Trendsceek utilizes a markov process to simulate the association between gene expression and cell coordinates, while SpatialDE is a Gaussian process regression-based method. SPARK identifies SVGs based on a spatial generalized linear mixed model with multiple spatial kernels, directly modeling spatial count data. BinSpect, a Gitto package, first creates a spatial grid using Delaunay to represent the associations between cells. For each input gene, SVGs are identified through K-means clustering. Giotto, as an integrative tool, combines the above four methods for identifying spatial genes and provides improvements in speed. Li et al. [63] compared different SVGs identification methods.

### Cell type annotation

Spatial transcriptomics annotation of cell types can be determined through specific spatial clusters of SVGs. However, this method is often not very accurate because sequencing-based spatial transcriptomics technologies usually do not achieve single-cell resolution, resulting in the possibility of more than one cell type per spot. The process of identifying and quantifying the relative contributions of each cell type and gene within a spot is known as deconvolution. Several tools have been developed to perform this task, typically requiring integration with scRNA-seq references. These methods include SPOTlight [64], SpatialDWLS [65], Cell2location [66], CellTrek [67], RCTD [68], DSTG [69], GraphST [70], Tangram [71], and STRIDE [72].

SPOTlight is a tool that provides an non-negative matrix factorization regression (NMFreg)-based model for deconvolution of mixed cells from a single-cell reference [64]. SpatialDWLS determines the cell type composition for each spatial location by applying damped least squares to infer the fraction for each selected cell type from spatial transcriptomics data [65]. spatialDWLS contains an additional filtration step to remove unrelated cell types, which is better in terms of precision. Cell2location is a Bayesian model-based tool that can parse fine-grained cell types in spatial transcriptomics data and create comprehensive cell maps of different tissues [66]. CellTrek employs co-embedding and metric learning methods combined with spatial transcriptomics and scRNA-seq data to infer the distribution and relative abundance of cell types within tissues [67]. RCTD combines sparse matrix factorization and tensor decomposition techniques to identify and quantify cell types and their relative spatial positions within tissues [68]. DSTG [69] deconvolves spatial transcriptomics data via graph-based convolutional networks to accurately deconvolve the gene expression observed at each point and restore its cellular structure. GraphST is a self-supervised contrast learning method that combines graph neural networks with self-supervised contrast learning to learn informative and discriminative spot representations by minimizing the embedding distance between spatially adjacent spots [70]. Tangram utilizes graph convolutional neural networks and modal adversarial training methods for spatial localization and deconvolution of cell types [71]. STRIDE is a deep learning-based method that integrates spatial transcriptomics and scRNA-seq data for cell type identification and deconvolution of spatial distribution [72]. Previous studies have conducted comprehensive benchmarking of common deconvolution methods, serving as valuable references for selection [1].

### Spatial trajectory inference

Cell trajectory analysis reconstructs the changing trajectories of cells over time by analyzing dynamic changes in gene expression between cells. Common spatial trajectory inference methods include StLearn [55] and SPATA [73]. StLearn [55] offers an algorithm called Pseudo-Spatial-Time (PST) trajectory analysis, which visualizes spatial trajectories in tissue sections and infers biological processes from gradients of transcriptional states across tissues. SPATA [73], on the other hand, analyzes dynamic changes using monocle3 to infer transcriptional patterns controlled by spatial organization dynamics.

### Cellular interaction

Cells within tissues constantly interact with each other, and identifying the ligand-receptor network between adjacent cells in TME is critical to understanding the drivers of tumorigenesis. Spatial transcriptomics enables the analysis of intercellular interactions at specific spatial locations. The Gene Graph Convolutional Neural Network (GCNG) [74] method transforms spatial transcriptomcis data into a relational graph between cells, convolving the graph with gene expression information to infer gene interactions involved in cell communication. SpaOTsc [75] reconstructs spatial cellular dynamics within tissues by establishing a graph between spatial transcriptomics and scRNA-seq datasets. MISTy [76] simulates interactions between labeled genes in neighboring cells by combining views from different spatial contexts. spaCI is an adaptive graph model that leverages spatial locations and gene expression profiles to identify ligand-receptor interactions in imaging-based spatial transcriptomics data [77]. It utilizes attention mechanisms and triplet loss to accurately infer cellular communications. Compared to other methods, spaCI excels in capturing complex cellular interactions and outperforms in identifying upstream transcriptional factors involved in active ligand-receptor signaling pathways.

### Drug discovery

The failure of cancer treatment is often attributed to tumor heterogeneity. While spatial transcriptomics provides increasingly detailed descriptions of heterogeneous tumor cells, applying this knowledge to clinical drug resistance research remains challenging. Beyondcell is a methodology designed to identifiy drug vulnerabilities using spatial transcriptomics data. It calculates a Beyondcell Score for each spot and drug pair, ranging from 0 to 1, to measure drug susceptibility. Spots are then classified into therapeutic clusters, and scores are visualized to understand the therapeutic architecture of the sample. Beyondcell aids in identifying potential drug effects across different cell types, facilitating precision medicine [78]. SpaRx utilizes graph-based domain adaptation to uncover spatial cellular response diversity to drugs by leveraging pharmacogenomic profiles. Through hybrid learning with dynamic adversarial adaptation, SpaRx accurately identifies spatial therapeutic variability, reveals molecular mechanisms of drug resistance, and identifies personalized drug targets and effective combinations [79].

## Integration of spatial transcriptomics with other omics

Spatial transcriptomics, while adept at deciphering cellular spatial expression changes and interactions at the RNA level, faces limitations due to the intricate molecular network within cells, comprised of nucleic acids, proteins, and various other small molecules. Only spatial transcriptome cannot fully encapsulate the entirety of molecular regulatory networks in cells. Hence, integrating spatial transcriptome with other omics data can better unveil cellular changes at spatial positions.

Combining spatial transcriptomics with proteomics is currently the most common research approach. Proteomics not only validates transcriptomic analysis results at the protein level but also can aid in cell annotation. For example, Liu et al. integrated spatial transcriptome and spatial proteome to determine the interaction between APOE^+^/CD163^+^ TAMs and EMT tumor cells at both the gene and protein levels [80]. Greenwald et al. used co-detection-by-indexing (CODEX) to help accurately annotating cell types of 10X Visium [81]. Besides, some other omics can also help explain the analysis results of spatial transcriptomics from different perspectives. For instance, Hu et, al. performed spatial transcriptomics and metabolomics profiling and found the intratumoral heterogeneity of clear cell renal cell carcinoma and a potential correlation between pyrimidine derivates and TILs [82]. Similarly, Cheng et, al. found that the energy metabolism of the tumor margin in oral squamous cell carcinoma was more active, and the cells in the tumor margin produced more ATP to address the higher metabolic costs for invasion using spatial metabolomics and the spatial transcriptomics [83].

## Applications of spatial transcriptomics sequencing technology in cancer research

**Fig. 4:**
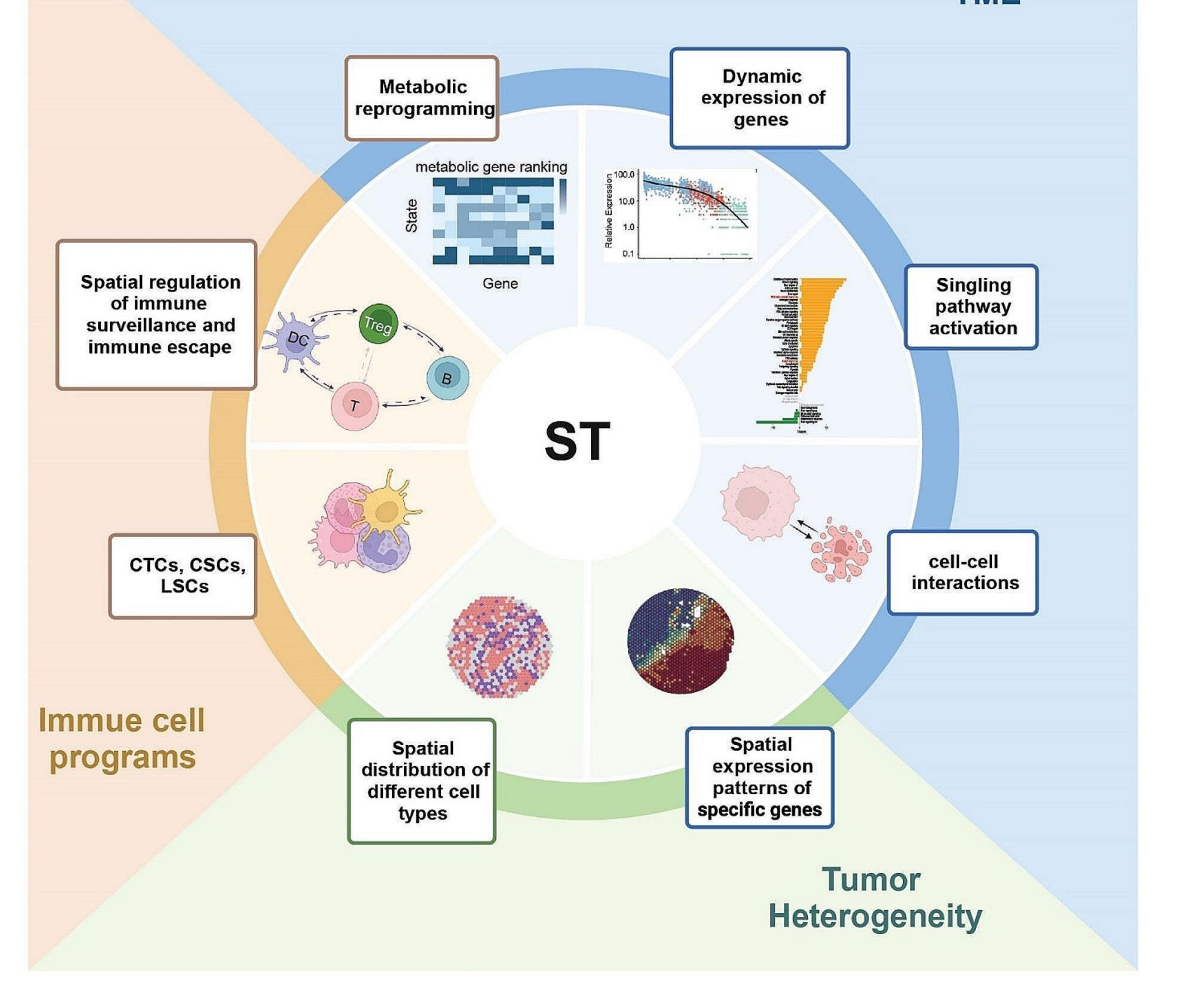
Application of spatial transcriptomics in cancer research

Spatial transcriptomics has myriad applications in cancer research, ranging from unraveling intra-tumoral heterogeneity and understanding the tumor-stroma crosstalk to identifying spatially regulated biomarkers (Fig. 4). The ability to study the spatial dynamics of gene expression within tumors has profound implications for advancing our understanding of tumor initiation, progression, therapeutic resistance, and the development of targeted and personalized treatment strategies.

Rather than being homogeneous entities, tumors are composed of distinct cell types, each contributing to the overall structure and behavior of the tumor. This results in significant heterogeneity among different tumors, posing challenges for both tumor diagnosis and treatment. Therefore, dissecting tumor cells and the microenvironment at the cellular spatial level to understand their heterogeneity can help reveal the spatial distribution of different cell types within tumor tissues and their specific spatial patterns of gene expression. Ultimately, this can help us understand how the spatial organization of cells affects tumor progression, treatment response, and drug resistance.

Spatial transcriptomics is a technology that precisely reveals the composition and distribution of different cell types within a tissue sample. Using this technology can facilitate the understanding of the types and states of cells surrounding the tumor as well as their interactions with tumor cells, and thereby reveal the complexity of the tumor microenvironment (TME). Indeed, some existing studies have mapped spatial transcriptomics landscapes of various cancers and confirmed the crucial role of TME in clinical diagnosis, disease progression, and treatment response, including the emergence of treatment resistance in tumors. Here, we summarize key findings using spatial transcriptomics on tumor heterogeneity and changes in the TME during different progressions in various tumors. The summaries highlight how this technique helps understand specific cancer types, identify new therapeutic targets, and improve clinical outcomes, and also highlight its transformative impact on reshaping the landscape of tumor biology and its implications for precision medicine.

### Lung cancer

Lung cancer is one of the leading causes of cancer-related deaths globally and a complex disease with numerous subtypes, each presenting different clinical outcomes. By utilizing spatial transcriptomics to compare the differences between subtypes at the cellular level, researchers can uncover the specific TMEs unique to each subtype, offering valuable insights for diagnosis and treatment. Wang et al. used spatial transcriptomics to compare the spatial heterogeneity of macrophages in different histological subtypes of lung cancer, ultimately discovering differences in the composition and spatial distribution of macrophages in each subtype [84]. Similarly, Xie et al. integrated spatial transcriptomics and scRNA-seq to evaluate the characteristic spectrum of squamous and glandular histological subtypes in lung adenocarcinoma (LUAD) patients, discovering the important role of endothelial cells (ECs) in the transition from a squamous to glandular pattern in the TME [85].

In addition, spatial transcriptomics has also been utilized to analyze specific or rare types of lung cancer. Wang et al. integrated scRNA-seq and spatial transcriptomics to study the cellular composition and spatial structure of multiple primary lung cancer (MPLC). They ultimately discovered a MPLC-specific subpopulation of AT2 cells [84]. In another study, Szeitz et al. conducted proteomic and spatial transcriptomics analyses of ALK-rearranged LUAD, revealing key factors contributing to both inter-tumor and intra-tumor heterogeneity [86]. These studies provide valuable insights into the spatial heterogeneity of lung cancer. In addition, there have been studies specifically focusing on small cell lung cancer (SCLC). Wu et al. employed a combination of spatial transcriptomics and multi-regional scRNA-seq to comprehensively characterize the transcriptional landscape of both SCLC tumor cells and the adjacent TME [87]. Meanwhile, Yang et al. extensively characterized the immune microenvironment in SCLC by integrating transcriptomics and protein profiling, identifying two distinct disease subtypes: immune-enriched (IE-subtype) and immune-deprived (ID-subtype) of SCLC [88]. By examining the spatial microenvironment of the tumor using spatial transcriptomics techniques, these studies challenged the conventional belief that SCLC is a homogeneous disease.

Besides, spatial transcriptomics is a powerful tool to reveal the cellular mechanisms underlying the differences in immune therapy among patients with lung cancer. The spatial transcriptomics research by Larroquette et al. identified a class of macrophages closely related to the immunotherapy of non-small cell lung cancer, and revealed the molecular mechanisms involved [89]. While Monkman et al. observed that the interaction between CD68^+^ macrophages and PD1^+^, FOXP3^+^ cells is especially enriched in immunotherapy-refractory tumors [90], and emphasized that the stromal region exhibits more distinct genetic information among different therapeutic groups undergoing immunotherapy [91].

### Colorectal cancer

Colorectal cancer (CRC) exhibits significant heterogeneity, leading to variable responses to treatment. Spatial transcriptomics can provide a spatially resolved cellular analysis of CRC with high precision. For example, Ozato et al. combined CRC spatial transcriptomics and scRNA-seq and discovered the localization of CRC cells and their cross-talking with co-localized leukocytes, which imparts them with anti-tumor immune, proliferative, and invasive properties [92]. Similarly, Qi et al. discovered the co-existence of FAP^+^ fibroblasts and SPP1^+^ macrophages using spatial transcriptomics [93]. Using spatial transcriptomics, Wood et, al. uncovered heterogeneity between patients, between matched lesions in the same patient, and within individual lesions of CRC [94]. In addition, Roelands et, al. demonstrated the heterogeneity of myeloid cells and macrophage populations between different tumorigenic stages of CRC [95].

Spatial transcriptomics is also used to study the differences between different subtypes of CRC. Pelka et al. utilized GeoMx DSP to study two distinct genetic subtypes of primary CRC and identified differences in the immune landscape between MMRd and MMRp tumors, elucidating cell-cell interaction networks within the spatial organization [96]. While Peng et, al. profiled the tumor heterogeneity landscape and identified two distinct types of cancer-associated fibroblasts (CAF) in CRC, of which inflammatory-cancer-associated fibroblasts (iCAF) have extensive crosstalk between stromal components in the TME and ultimately promote tumor progression and metastasis [97].

As tumor metastasis is the most lethal cause of CRC, spatial transcriptomics is also used by many researchers to investigate the cellular and molecular alterations that occur during CRC metastasis. Wang et al. charted the spatial cellular landscape of CRC and well-matched liver metastatic CRC using scRNA-seq and spatial transcriptomics [98]. They found the enrichment of distinct fibroblast subtypes in the primary tumor and liver metastatic lesions, respectively. In another study, Wu et, al. found that the immune microenvironment has undergone extensive spatiotemporal remodeling into an immunosuppressive status from primary tumor to liver metastasis [99]. Peculiarly, Li et, al. performed scRNA-seq and spatial transcriptomics analysis in primary CRC and metastases in the liver (lCRC) or ovary (oCRC), ultimately identifying a stem-like cell cluster as the cause of CRC liver and ovarian metastasis [100].

### Liver cancer

Primary liver cancer (PLC) ranks as the second most lethal neoplasm and is mainly subdivided into hepatocellular carcinoma (HCC), intrahepatic cholangiocarcinoma (ICC), and combined hepatocellular-cholangiocarcinoma and intrahepatic cholangiocarcinoma (cHCC-ICC), whose etiology and biological diversity contribute to the high degree of heterogeneity observed in PLC tumors [101]. Several studies have applied spatial transcriptomics to provide insights into the spatial heterogeneity of PLC. Zhou et al. combined scRNA-seq, spatial transcriptomics, and bulk multi-omics to elaborate the cellular and molecular structures of the three PLC types at the single-cell and spatial levels, describing the microenvironmental differences among the three molecular subtypes [102]. In another study, machine learning was combined with spatial transcriptomics and a comprehensive deep learning-based phenotypic analysis was performed for the three subtypes of PLC to improve their treatment decisions and ultimate clinical outcomes [103]. Besides, Gan et al. used spatial transcriptomics to study cHCC-CCA, a rare PLC type, and found that the cHCC-CCA-specific tertiary lymphoid structure (TLS) gene set is associated with high-intensity immune infiltration [104].

To date, there are few effective non-surgical strategies for treatment PLC, and there is a lack of specific drug targets for effective therapeutic intervention [105]. By describing the TME, spatial transcriptomics provides new insights into the pathogenesis, development, diagnosis, and treatment of PLC. Wu et al. conducted spatial transcriptomics analysis on tumor tissues of PLC patients using 10X Visium, unveiling the relationship between TME remodeling and tumor metastasis through the distribution of PROM1^+^ and CD47^+^ cancer stem cells, offering new insights for tumor intervention [106]. Liu and colleagues integrated spatial transcriptomics and scRNA-seq to delineate the spatial architecture of the tumor immune barrier (TIB). Their investigation revealed that the TIB, formed through the interactions between SPP1^+^ macrophages and CAFs, is associated with immunotherapy efficacy. By disrupting the SPP1-mediated TIB structure, the effectiveness of immune checkpoint blockade in treating HCC was enhanced [107].

Additionally, employing the 10X Visium platform, Wang et al. identified malignant subgroups within the HCC TME and discovered an enrichment of CCL15 in the tumor core regions, implicating the establishment of an immunosuppressive microenvironment [108]. Wu and colleagues utilized Stereo-seq to analyze tumor tissues from patients with PLC, defining a 500 μm wide area centered on the tumor margins as the invasion zone. Within this zone, significant immunosuppression, metabolic reprogramming, and hepatocyte damage were identified [109]. Chew also reported the discovery of invasive areas around tumor margins in liver cancer patients using Stereo-seq, characterized by the overexpression of serum amyloid A1 and A2, which correlates with immunosuppression and poor prognosis [110].

Furthermore, spatial transcriptomics has been employed to investigate the spatiotemporal evolution of metastatic and primary hepatocellular carcinoma. Sun and colleagues utilized the NanoString GeoMx platform for whole transcriptome analysis (WTA) with spatial resolution to characterize molecular alterations at various metastatic sites and time points elucidating the dynamic processes of tumor evolution [111].

### Breast cancer

Breast cancer (BC) is the most common cancer in women and has long been a threat to women’s lives and health [112]. It is classified into several types, primarily based on where the disease begins in the breast, the type of cells involved, and their hormone receptor status. Common BC types include ductal carcinoma in situ (DCIS), invasive ductal carcinoma (IDC), and invasive lobular carcinoma (ILC). Based on hormone receptor status (estrogen receptor [ER] or progesterone receptor [PR]) and human epidermal receptor 2 (HER2) status, BC can be divided into subtypes of hormone receptor-positive (ER^+^/PR^+^) BC, HER2^+^ BC, and triple-negative (TNBC, ER^−^/PR^−^/HER2^−^) BC [113].

DCIS is an early form of breast cancer that rarely progresses to more malignant IDC [114]. However, these patients are often overtreated based on current clinical standards. Casasent et al. revealed the genomic lineage relationships between in situ and invasive tumor subgroups by analyzing heterogeneous populations of ductal carcinoma through genomic analysis [115]. Spatial transcriptomics showed that DCIS with GATA3 mutations enhances epithelial-mesenchymal transition and angiogenesis, indicating early genetic alterations in malignancy. GATA3 mutations serve as potential markers to differentiate between high-risk and low-risk DCIS. This finding was corroborated by Nagasawa et al., who identified GATA3 mutations as potential recurrence indicators and discovered downregulation of the progesterone receptor, offering new insights into DCIS classification and treatment optimization [116].

TNBC is the most aggressive BC subtype and the most difficult to treat clinically [117]. Bassiouni et al. conducted spatial transcriptomics analysis on 28 tissue sections from 14 TNBC patients, revealing diverse transcriptomics substructures within tumor sections. The comprehensive analysis of all samples identified nine distinct transcriptional subgroups with varying functional roles and prognostic implications [118]. Additionally, the study uncovered non-random, directed spatial dependencies between shared transcriptional subgroups through connective count analysis, demonstrating conserved spatial transcriptional architectures in TNBC. This research significantly contributes to our understanding of TNBC’s internal heterogeneity and disease progression.

The high heterogeneity of breast cancer results in varying responses to different treatments, and spatial transcriptomics can help unravel the underlying reasons at the cellular spatial level. Through spatial transcriptomics studies, Donati and colleagues found that the spatial distribution of immune cells in TNBC affects resistance to neoadjuvant chemotherapy (NAC) [119]. These findings open new avenues for improving TNBC treatment strategies and personalizing therapy. In addition, Tashireva et al. investigated differences in the spatial distribution of the TME in PD-L1-negative and PD-L1-positive TNBC, highlighting the prevalence of PD1-negative M2 macrophages and PD1-negative T lymphocytes in PD-L1-positive tumors [120]. This may be the underlying reason why patients with TNBC do not benefit from ICI. In another study [121], TNBC was further categorized into homologous recombination deficiency (HRD) group and non-HRD group. HRD tumors, known for their higher mutation burden and immunogenicity, are considered biomarkers for ICI response, yet the actual response in TNBC to ICIs is more complex. The authors utilized single-cell and spatial transcriptomics to compare the TME of both TNBC groups and found that HRD TME has fewer dysfunctional immune phenotypes but not higher immune cell content. Combining HRD status with methods assessing immune cell content may better predict ICI response in TNBC.

In summary, spatial transcriptomics plays a crucial role in the BC classification, diagnosis, and treatment.

### Pancreatic cancer

Pancreatic ductal adenocarcinoma (PDAC) represents an exceptionally lethal malignancy, accounting for over 90% of all pancreatic cancer cases [122]. Despite this significant burden, effective therapeutic strategies remain elusive.

In 2020, Reuben Moncada’s team mapped the spatial transcriptomics of PDAC for the first time, revealing the spatially restricted enrichments and distinct co-enrichments of subpopulations of ductal cells, macrophages, dendritic cells, and cancer cells [123]. Subsequently, other researchers used spatial transcriptome to determine the histological characteristics of different subpopulations within the TME of PDAC [124]. These spatial transcriptomics landscapes provide a basis for understanding the intricate substructure of PDAC. Based on the PDAC TME landscape, some studies have further analyzed specific cell populations in the TME.

Given the poor clinical prognosis and notably poor response to immunotherapy in PDAC, a more nuanced understanding of the immunological microenvironment is essential. Yousuf and colleagues have used spatially resolved multi-omics single-cell analysis and delineated a comprehensive landscape of the immune milieu in PDAC, uncovering the multifaceted immune dysfunction inherent to the disease [125]. This work provides novel insights into the functional investigation of PDAC and the exploration of actionable therapeutic targets. Moncada have identified the colocalization of inflammatory fibroblasts and cancer cells expressing a stress-response gene module, highlighting the significant role of fibroblasts in the progression of PDAC [123]. Consequently, another study shifted the focus to targeting the stromal cells. Croft et al. combined spatial transcriptomics with scRNA-seq to compare the spatial distribution and gene signatures of CAFs within PDAC tumors [126]. Survival analysis revealed that tumor-proximal CAFs exhibit increased characteristics associated with a poorer prognosis compared to tumor-distal CAFs.

Perineural invasion (PNI), a hallmark of PDAC characterized by cancer cells infiltrating the perineural space leading to neuropathy [127]. The presence of PNI indicates a greater probability of local recurrence and metastasis. To unveil the effects of Schwann cells, the most prevalent cell type in peripheral nerves, on the neuro-stroma niche, Xue et al. performed scRNA-seq and microarray-based spatial transcriptomics analysis of PDAC tissues. Their results suggested that Schwann cells might drive tumor cells and CAFs towards more malignant subtypes: basal-like and iCAFs [128]. In another study, Weitz and colleagues employed GeoMx DSP to investigate PDAC during PNI and uncovered that PDAC induces neural damage and promotes transcriptional and functional reparations in the surrounding glial cells during PNI [129]. These findings offer novel insights into the neuropathic phenomena within PDAC.

Most PDACs recur after resection. In a study on PDAC recurrence [130], the authors conducted spatial analyses of immune pathway-related genes and proteins within tumor tissues of PDAC patients with various recurrence sites. It was discovered that non-recurrent PDACs exhibit high immunogenicity and adaptive immunity and are enriched in pro-inflammatory chemokines, whereas PDACs with different recurrence patterns have distinct inflammatory/stromal responses, potentially influencing infiltration patterns and patient prognosis. These findings may contribute to personalized treatment approaches for PDAC.

### Glioblastoma

Recurrence is one of the main clinical challenges faced by glioblastoma (GBM), making it crucial to decipher the mechanisms of recurrence to identify intervention targets. By integrating single-cell and spatial transcriptomics, Andrieux et al. uncovered the molecular characteristics and spatial distribution of a specific cell subtype associated with recurrence in GBM, known as infiltrative 5ALA^+^ cell [131]. Wang et al. performed spatial analyses of paired primary and recurrent samples from patients receiving standard-of-care therapy for GBM and found changes in tumor signaling pathways and the microenvironment with targetable potential [132]. Additionally, Loussouarn et al. examined immune markers in primary and recurrent GBM patients and found that the distribution of immune markers in these tumors is extremely heterogeneous, which could explain why GBM is refractory to universal immunotherapies [133].

As GBM tumors are usually refractory to immune checkpoint therapy (ICT). Only a minority of patients with GBM respond to immunotherapy, and always only partially. Utilizing spatial transcriptomics to analyze the TME helps reveal the reasons for the ineffectiveness of tumor immunotherapy. For example, Goswami et al. identified selective expression of KDM6B in intertumoral myeloid cell subsets in GBM tumors and their impact on anti-PD1 efficacy [134]. Mei et al. identified unique monocyte-derived tumor-associated macrophage subpopulations with functional plasticity that highly expressed the immunosuppressive SIGLEC9 gene and preferentially accumulated in nonresponders to anti-PD-1 treatment [135]. Ravi et al. applied an in-silico multidimensional model integrating spatially resolved and single-cell gene expression data of 45,615 immune cells from 12 tumor samples and identified that a subset of interleukin-10-releasing HMOX1^+^ myeloid cells, spatially localized to mesenchymal-like tumor regions, drive T-cell exhaustion and thus contribute to the immunosuppressive TME [136].

## Limitations and challenges of spatial transcriptomics

Spatial transcriptomics introduces spatial information into transcriptomics sequencing, which makes it possible to study the spatial expression distribution of genes in various tissues and organs. However, as a new technique, the current spatial transcriptome still has some defects.

First, the abundance of RNAs captured by the spatial transcriptomics is still very limited. Image-based spatial transcriptomics techniques like seqFISH can only localize hundreds to thousands of genes in intact tissue but not whole transcriptomics scale. Although sequencing-based spatial transcriptomics techniques can theoretically perform whole transcriptomics sequencing, due to the probe capture efficiency, the transcripts that can be detected is also limited, and the depth and coverage of spatial transcriptomics are far from the bulk transcriptomics. Limited sequencing depth and coverage make it hard to detect those genes that are relatively less expressed. At the same time, because the resolution of technologies such as 10X is only about 100 μm, a spot contains multiple cells, which makes it impossible to achieve single-cell resolution. This makes it particularly difficult to study immune cells solely using the spatial transcriptomics.

Second, preparing samples for spatial transcriptomics is laborious. During the sample preparation process, it typically involves sample slicing, tissue dissociation, and the connection of multiple barcodes and indexes. Additionally, optimization steps such as tissue permeabilization may be necessary to find the optimal conditions. The complexity of the preparation process increases experimental costs, resulting in spatial transcriptomics costing several times more than bulk transcriptomics.

Last, data interpretation of spatial transcriptomics poses challenges. Due to massive amount of data generated from spatial transcriptomics, there are huge demands on computational power and storage capacity. Additionally, the analysis workflow for spatial transcriptomics data is more complex, often relying on sophisticated statistical models. Furthermore, interpreting the data requires a deeper understanding of cellular biology and immunology. Thus, spatial transcriptomics data analysis requires analysts to have a strong foundation in statistics, cellular biology, and immunology. Moreover, current sequencing depth and resolution of spatial transcriptomics are not ideal, leading to almost ubiquitous missing values in each sequencing sample, making spot annotation and gene expression analysis more difficult during data analysis.

## Future development and prospects of spatial transcriptomics sequencing

Rapid advances of spatial transcriptomics technology have made it possible to characterize the cellular landscape of tumors while retaining spatial location information, so spatial transcriptomics technology is a powerful tool to understand cell functions and their interactions, provide unprecedented insights into tumor heterogeneity and microenvironment. By gaining a deeper understanding of the interactions between tumors and their surrounding environment, we could develop and optimize precision treatment strategies to improve effectiveness.

However, spatial transcriptomics technologies need to be improved in different aspects. First, it is urgent to develop novel sequencing technologies that are more sensitive, provide broader tissue coverage, offer higher spatial resolution, and are more mature and cost effective. Second, developing powerful computational tools and algorithms to analyze spatial transcriptomics data is a major challenge in the field. Third, spatial transcriptomics data are surging at an unprecedented rate, so it is important to build specialized databases to summarize and integrate such huge amounts of data from different studies and different platforms. Fourth, current spatial transcriptomics technologies mainly focus on detecting mRNA molecules with polyA tails, developing improved technology measuring different RNA molecules such as lncRNA, circRNA and miRNA will provide much more comprehensively information. With the improvement of spatial transcriptomics technologies and analytic methods, the deeper understanding of tumorigenesis and development is expected to identify new diagnostic markers and therapeutic targets.

## Data Availability

No datasets were generated or analysed during the current study.

## Abbreviations

BC: Breast cancer
bsAb: Bispecific Antibody
CAF: Cancer-Associated Fibroblast
cDNA: Complementary DNA
cHCC-ICC: Combined Hepatocellular-Cholangiocarcinoma and Intrahepatic Cholangiocarcinoma
CRC: Colorectal Cancer
DCIS: Ductal Carcinoma In Situ
EC: Endothelial Cell
ER: Estrogen Receptor
ExSeq: Expansion Sequencing
FISH: Fluorescence In Situ Hybridization
FISSEQ: Fluorescence In Situ Sequencing
GBM: Glioblastoma
GCN: Graph: Convolutional Networks
GeoMx DSP: GeoMx Digital Spatial Profiling
GEO-seq: Geographical Position Sequencing
HCC: Hepatocellular Carcinoma
HDST: High-definition Spatial Transcriptomics
HER2: Human Epidermal Receptor 2
HRD: Homologous Recombination Deficiency
HybISS: Hybridization-based in situ sequencing
iCAF: Inflammatory-Cancer-Associated Fibroblast
ICC: Intrahepatic Cholangiocarcinoma
ICI: Immune Checkpoint Inhibitor
ICT: Immune Checkpoint Therapy
IDC: Invasive Ductal Carcinoma
ILC: Invasive Lobular Carcinoma
ISH: In Situ Hybridization
ISS: In Situ Sequencing
LCM: Laser Capture Microdissection
Lock-Seq: Lock-probe in situ sequencing
MERFISH: Multiplexed Error-Robust Fluorescence In Situ Hybridization
MPLC: Multiple Primary Lung Cancer
mRNA: messenger RNA
NAC: Neoadjuvant Chemotherapy
NMFreg: Non-negative Matrix Factorization regression
osmFISH: Ouroboros single-molecule Fluorescence In Situ Hybridization
PDAC: Pancreatic Ductal Adenocarcinoma
PLC: Primary Liver Cancer
PNI: Perineural Invasion
PR: Progesterone Receptor
PST: Pseudo-Spatial-Time
RCA: Rolling-Circle Amplification
RNA-seq: RNA sequencing
SBH: Sequence-by-hybridization
SBL: Sequence-by-ligation
scDNA-seq: Single-cell: DNA sequencing
SCLC: Small Cell Lung Cancer
scRNA-seq: Single-cell: RNA sequencing
seqFISH: Sequential: Fluorescence In Situ Hybridization
smFISH: Single-molecule Fluorescence In Situ Hybridization
SVG: Spatially Variable Gene
TAM: Tumor-Associated Macrophage
TIB: Tumor Immune Barrier
TIVA: Transcriptome In Vivo Analysis
TLS: Tertiary Lymphoid Structure
TME: Tumor Microenvironment
TNBC: Triple-Negative Breast Cancer
TSCS: Topographic Single Cell Sequencing
UMI: Unique Molecular Indexe
WGA: Whole-Genome Amplification
WTA: Whole Transcriptome Analysis

## References

[1] Li B, Zhang W, Guo C, Xu H, Li L, Fang M, Hu Y, Zhang X, Yao X, Tang M (2022). Benchmarking spatial and single-cell transcriptomics integration methods for transcript distribution prediction and cell type deconvolution. Nat Methods.

[2] Shapiro E, Biezuner T, Linnarsson S (2013). Single-cell sequencing-based technologies will revolutionize whole-organism science. Nat Rev Genet.

[3] Tian L, Chen F, Macosko EZ (2023). The expanding vistas of spatial transcriptomics. Nat Biotechnol.

[4] Emmert-Buck MR, Bonner RF, Smith PD, Chuaqui RF, Zhuang Z, Goldstein SR, Weiss RA, Liotta LA (1996). Laser capture microdissection. Science.

[5] Bonner RF, Emmert-Buck M, Cole K, Pohida T, Chuaqui R, Goldstein S, Liotta LA (1997). Laser capture microdissection: molecular analysis of tissue. Science.

[6] Nichterwitz S, Chen G, Aguila Benitez J, Yilmaz M, Storvall H, Cao M, Sandberg R, Deng Q, Hedlund E (2016). Laser capture microscopy coupled with Smart-seq2 for precise spatial transcriptomic profiling. Nat Commun.

[7] Chen J, Suo S, Tam PP, Han JJ, Peng G, Jing N (2017). Spatial transcriptomic analysis of cryosectioned tissue samples with Geo-Seq. Nat Protoc.

[8] Lovatt D, Ruble BK, Lee J, Dueck H, Kim TK, Fisher S, Francis C, Spaethling JM, Wolf JA, Grady MS (2014). Transcriptome in vivo analysis (TIVA) of spatially defined single cells in live tissue. Nat Methods.

[9] Medaglia C, Giladi A, Stoler-Barak L, De Giovanni M, Salame TM, Biram A, David E, Li H, Iannacone M, Shulman Z, Amit I (2017). Spatial reconstruction of immune niches by combining photoactivatable reporters and scRNA-seq. Science.

[10] Boisset JC, Vivie J, Grun D, Muraro MJ, Lyubimova A, van Oudenaarden A (2018). Mapping the physical network of cellular interactions. Nat Methods.

[11] Buongiorno-Nardelli M, Amaldi F (1970). Autoradiographic detection of molecular hybrids between RNA and DNA in tissue sections. Nature.

[12] Bauman JG, Wiegant J, Borst P, van Duijn P (1980). A new method for fluorescence microscopical localization of specific DNA sequences by in situ hybridization of fluorochromelabelled RNA. Exp Cell Res.

[13] Raj A, van den Bogaard P, Rifkin SA, van Oudenaarden A, Tyagi S (2008). Imaging individual mRNA molecules using multiple singly labeled probes. Nat Methods.

[14] Wang F, Flanagan J, Su N, Wang LC, Bui S, Nielson A, Wu X, Vo HT, Ma XJ, Luo Y (2012). RNAscope: a novel in situ RNA analysis platform for formalin-fixed, paraffin-embedded tissues. J Mol Diagn.

[15] Codeluppi S, Borm LE, Zeisel A, La Manno G, van Lunteren JA, Svensson CI, Linnarsson S (2018). Spatial organization of the somatosensory cortex revealed by osmFISH. Nat Methods.

[16] Lubeck E, Coskun AF, Zhiyentayev T, Ahmad M, Cai L (2014). Single-cell in situ RNA profiling by sequential hybridization. Nat Methods.

[17] Eng CL, Lawson M, Zhu Q, Dries R, Koulena N, Takei Y, Yun J, Cronin C, Karp C, Yuan GC, Cai L (2019). Transcriptome-scale super-resolved imaging in tissues by RNA seqFISH. Nature.

[18] Chen KH, Boettiger AN, Moffitt JR, Wang S, Zhuang X (2015). RNA imaging. Spatially resolved, highly multiplexed RNA profiling in single cells. Science.

[19] Moffitt JR, Bambah-Mukku D, Eichhorn SW, Vaughn E, Shekhar K, Perez JD, Rubinstein ND, Hao J, Regev A, Dulac C, Zhuang X. Molecular, spatial, and functional single-cell profiling of the hypothalamic preoptic region. Science 2018, 362. 10.1126/science.aau5324 PMC6482113 30385464

[20] Xia C, Fan J, Emanuel G, Hao J, Zhuang X (2019). Spatial transcriptome profiling by MERFISH reveals subcellular RNA compartmentalization and cell cycle-dependent gene expression. Proc Natl Acad Sci U S A.

[21] Ke R, Mignardi M, Pacureanu A, Svedlund J, Botling J, Wahlby C, Nilsson M (2013). In situ sequencing for RNA analysis in preserved tissue and cells. Nat Methods.

[22] Lee JH, Daugharthy ER, Scheiman J, Kalhor R, Ferrante TC, Terry R, Turczyk BM, Yang JL, Lee HS, Aach J (2015). Fluorescent in situ sequencing (FISSEQ) of RNA for gene expression profiling in intact cells and tissues. Nat Protoc.

[23] Lee JH, Daugharthy ER, Scheiman J, Kalhor R, Yang JL, Ferrante TC, Terry R, Jeanty SS, Li C, Amamoto R (2014). Highly multiplexed subcellular RNA sequencing in situ. Science.

[24] Gyllborg D, Langseth CM, Qian X, Choi E, Salas SM, Hilscher MM, Lein ES, Nilsson M (2021). Correction to ‘Hybridization-based in situ sequencing (HybISS) for spatially resolved transcriptomics in human and mouse brain tissue’. Nucleic Acids Res.

[25] Alon S, Goodwin DR, Sinha A, Wassie AT, Chen F, Daugharthy ER, Bando Y, Kajita A, Xue AG, Marrett K et al. Expansion sequencing: spatially precise in situ transcriptomics in intact biological systems. Science 2021, 371. 10.1126/science.aax2656 PMC7900882 33509999

[26] Wang X, Allen WE, Wright MA, Sylwestrak EL, Samusik N, Vesuna S, Evans K, Liu C, Ramakrishnan C, Liu J et al. Three-dimensional intact-tissue sequencing of single-cell transcriptional states. Science 2018, 361. 10.1126/science.aat5691 PMC6339868 29930089

[27] Stahl PL, Salmen F, Vickovic S, Lundmark A, Navarro JF, Magnusson J, Giacomello S, Asp M, Westholm JO, Huss M (2016). Visualization and analysis of gene expression in tissue sections by spatial transcriptomics. Science.

[28] Wang Y, Ma S, Ruzzo WL (2020). Spatial modeling of prostate cancer metabolic gene expression reveals extensive heterogeneity and selective vulnerabilities. Sci Rep.

[29] Vickovic S, Eraslan G, Salmen F, Klughammer J, Stenbeck L, Schapiro D, Aijo T, Bonneau R, Bergenstrahle L, Navarro JF (2019). High-definition spatial transcriptomics for in situ tissue profiling. Nat Methods.

[30] Rodriques SG, Stickels RR, Goeva A, Martin CA, Murray E, Vanderburg CR, Welch J, Chen LM, Chen F, Macosko EZ (2019). Slide-seq: a scalable technology for measuring genome-wide expression at high spatial resolution. Science.

[31] Stickels RR, Murray E, Kumar P, Li J, Marshall JL, Di Bella DJ, Arlotta P, Macosko EZ, Chen F (2021). Highly sensitive spatial transcriptomics at near-cellular resolution with Slide-seqV2. Nat Biotechnol.

[32] Cho CS, Xi J, Si Y, Park SR, Hsu JE, Kim M, Jun G, Kang HM, Lee JH (2021). Microscopic examination of spatial transcriptome using seq-scope. Cell.

[33] Srivatsan SR, Regier MC, Barkan E, Franks JM, Packer JS, Grosjean P, Duran M, Saxton S, Ladd JJ, Spielmann M (2021). Embryo-scale, single-cell spatial transcriptomics. Science.

[34] Chen A, Liao S, Cheng M, Ma K, Wu L, Lai Y, Qiu X, Yang J, Xu J, Hao S (2022). Spatiotemporal transcriptomic atlas of mouse organogenesis using DNA nanoball-patterned arrays. Cell.

[35] Merritt CR, Ong GT, Church SE, Barker K, Danaher P, Geiss G, Hoang M, Jung J, Liang Y, McKay-Fleisch J (2020). Multiplex digital spatial profiling of proteins and RNA in fixed tissue. Nat Biotechnol.

[36] Liu Y, Yang M, Deng Y, Su G, Enninful A, Guo CC, Tebaldi T, Zhang D, Kim D, Bai Z (2020). High-spatial-resolution multi-omics sequencing via Deterministic Barcoding in tissue. Cell.

[37] He S, Bhatt R, Brown C, Brown EA, Buhr DL, Chantranuvatana K, Danaher P, Dunaway D, Garrison RG, Geiss G (2022). High-plex imaging of RNA and proteins at subcellular resolution in fixed tissue by spatial molecular imaging. Nat Biotechnol.

[38] Axelrod S, Carr AJ, Freeman J, Ganguli D, Long B, Tung T. others: Starfish: Open Source Image Based Transcriptomics and Proteomics Tools. 2018.

[39] Perkel JM (2019). Starfish enterprise: finding RNA patterns in single cells. Nature.

[40] Hao Y, Hao S, Andersen-Nissen E, Mauck WM 3rd, Zheng S, Butler A, Lee MJ, Wilk AJ, Darby C, Zager M, et al. Integrated analysis of multimodal single-cell data. Cell. 2021;184:3573–e35873529. 10.1016/j.cell.2021.04.048 PMC8238499 34062119

[41] Stuart T, Butler A, Hoffman P, Hafemeister C, Papalexi E, Mauck WM 3rd, Hao Y, Stoeckius M, Smibert P, Satija R. Comprehensive Integration of Single-Cell Data. Cell. 2019;177:1888–902. e1821. 10.1016/j.cell.2019.05.031 PMC6687398 31178118

[42] Wolf FA, Angerer P, Theis FJ. SCANPY: large-scale single-cell gene expression data analysis. Genome Biol. 2018;19:15. 10.1186/s13059-017-1382-0 PMC5802054 29409532

[43] 10x Genomics. SpaceRanger [Internet]. Available from: https://www.10xgenomics.com/products/spaceranger

[44] Sanger Institute. Single-cell analysis workflows (SAW) [Internet]. Available from: https://www.sanger.ac.uk/tool/saw/

[45] Dries R, Zhu Q, Dong R, Eng CL, Li H, Liu K, Fu Y, Zhao T, Sarkar A, Bao F, et al. Giotto: a toolbox for integrative analysis and visualization of spatial expression data. Genome Biol. 2021;22:78. 10.1186/s13059-021-02286-2 PMC7938609 33685491

[46] Bergenstrahle J, Larsson L, Lundeberg J. Seamless integration of image and molecular analysis for spatial transcriptomics workflows. BMC Genomics. 2020;21:482. 10.1186/s12864-020-06832-3 PMC7386244 32664861

[47] Palla G, Spitzer H, Klein M, Fischer D, Schaar AC, Kuemmerle LB, Rybakov S, Ibarra IL, Holmberg O, Virshup I (2022). Squidpy: a scalable framework for spatial omics analysis. Nat Methods.

[48] Hafemeister C, Satija R (2019). Normalization and variance stabilization of single-cell RNA-seq data using regularized negative binomial regression. Genome Biol.

[49] Hong D, Balzano L, Fessler JA (2018). Asymptotic performance of PCA for high-dimensional heteroscedastic data. J Multivar Anal.

[50] Maaten, Lvd. Hinton GEJJoMLR: Visualizing Data using t-SNE. 2008, 9:2579–605.

[51] McInnes L, Healy JJA. UMAP: Uniform Manifold Approximation and Projection for Dimension Reduction. 2018, abs/1802.03426.

[52] Zhao E, Stone MR, Ren X, Guenthoer J, Smythe KS, Pulliam T, Williams SR, Uytingco CR, Taylor SEB, Nghiem P (2021). Spatial transcriptomics at subspot resolution with BayesSpace. Nat Biotechnol.

[53] Yang Y, Shi X, Liu W, Zhou Q, Chan Lau M, Chun Tatt Lim J, Sun L, Ng CCY, Yeong J, Liu J. SC-MEB: spatial clustering with hidden Markov random field using empirical Bayes. Brief Bioinform 2022, 23. 10.1093/bib/bbab466 PMC8690176 34849574

[54] Hu J, Li X, Coleman K, Schroeder A, Ma N, Irwin DJ, Lee EB, Shinohara RT, Li M (2021). SpaGCN: integrating gene expression, spatial location and histology to identify spatial domains and spatially variable genes by graph convolutional network. Nat Methods.

[55] Pham D, Tan X, Balderson B, Xu J, Grice LF, Yoon S, Willis EF, Tran M, Lam PY, Raghubar A (2023). Robust mapping of spatiotemporal trajectories and cell-cell interactions in healthy and diseased tissues. Nat Commun.

[56] Cheng A, Hu G, Li WV. Benchmarking cell-type clustering methods for spatially resolved transcriptomics data. Brief Bioinform 2023, 24. 10.1093/bib/bbac475 PMC9851325 36410733

[57] Xu C, Jin X, Wei S, Wang P, Luo M, Xu Z, Yang W, Cai Y, Xiao L, Lin X (2022). DeepST: identifying spatial domains in spatial transcriptomics by deep learning. Nucleic Acids Res.

[58] Dong K, Zhang S (2022). Deciphering spatial domains from spatially resolved transcriptomics with an adaptive graph attention auto-encoder. Nat Commun.

[59] Tang Z, Li Z, Hou T, Zhang T, Yang B, Su J, Song Q (2023). SiGra: single-cell spatial elucidation through an image-augmented graph transformer. Nat Commun.

[60] Edsgard D, Johnsson P, Sandberg R (2018). Identification of spatial expression trends in single-cell gene expression data. Nat Methods.

[61] Svensson V, Teichmann SA, Stegle O (2018). SpatialDE: identification of spatially variable genes. Nat Methods.

[62] Sun S, Zhu J, Zhou X (2020). Statistical analysis of spatial expression patterns for spatially resolved transcriptomic studies. Nat Methods.

[63] Li K, Yan C, Li C, Chen L, Zhao J, Zhang Z, Bao S, Sun J, Zhou M (2022). Computational elucidation of spatial gene expression variation from spatially resolved transcriptomics data. Mol Ther Nucleic Acids.

[64] Elosua-Bayes M, Nieto P, Mereu E, Gut I, Heyn H (2021). SPOTlight: seeded NMF regression to deconvolute spatial transcriptomics spots with single-cell transcriptomes. Nucleic Acids Res.

[65] Dong R, Yuan GC (2021). SpatialDWLS: accurate deconvolution of spatial transcriptomic data. Genome Biol.

[66] Kleshchevnikov V, Shmatko A, Dann E, Aivazidis A, King HW, Li T, Elmentaite R, Lomakin A, Kedlian V, Gayoso A (2022). Cell2location maps fine-grained cell types in spatial transcriptomics. Nat Biotechnol.

[67] Wei R, He S, Bai S, Sei E, Hu M, Thompson A, Chen K, Krishnamurthy S, Navin NE (2022). Spatial charting of single-cell transcriptomes in tissues. Nat Biotechnol.

[68] Cable DM, Murray E, Zou LS, Goeva A, Macosko EZ, Chen F, Irizarry RA (2022). Robust decomposition of cell type mixtures in spatial transcriptomics. Nat Biotechnol.

[69] Song Q, Su J. DSTG: deconvoluting spatial transcriptomics data through graph-based artificial intelligence. Brief Bioinform 2021, 22. 10.1093/bib/bbaa414 PMC8425268 33480403

[70] Long Y, Ang KS, Li M, Chong KLK, Sethi R, Zhong C, Xu H, Ong Z, Sachaphibulkij K, Chen A (2023). Spatially informed clustering, integration, and deconvolution of spatial transcriptomics with GraphST. Nat Commun.

[71] Biancalani T, Scalia G, Buffoni L, Avasthi R, Lu Z, Sanger A, Tokcan N, Vanderburg CR, Segerstolpe A, Zhang M (2021). Deep learning and alignment of spatially resolved single-cell transcriptomes with Tangram. Nat Methods.

[72] Sun D, Liu Z, Li T, Wu Q, Wang C (2022). STRIDE: accurately decomposing and integrating spatial transcriptomics using single-cell RNA sequencing. Nucleic Acids Res.

[73] Kueckelhaus J, Ehr Jv, Ravi VM, Will P, Joseph K, Beck J, Hofmann UG, Delev D, Schnell O, Heiland DH. Inferring spatially transient gene expression pattern from spatial transcriptomic studies. 2020:2020.2010.2020.346544.

[74] Yuan Y, Bar-Joseph Z (2020). GCNG: graph convolutional networks for inferring gene interaction from spatial transcriptomics data. Genome Biol.

[75] Cang Z, Nie Q. Inferring spatial and signaling relationships between cells from single cell transcriptomic data. *Nat Commun* 2020, 11:2084. 10.1038/s41467-020-15968-5 PMC7190659 32350282

[76] Tanevski J, Flores ROR, Gabor A, Schapiro D, Saez-Rodriguez J (2022). Explainable multiview framework for dissecting spatial relationships from highly multiplexed data. Genome Biol.

[77] Tang Z, Zhang T, Yang B, Su J, Song Q. spaCI: deciphering spatial cellular communications through adaptive graph model. Brief Bioinform 2023, 24. 10.1093/bib/bbac563 PMC9851335 36545790

[78] Fustero-Torre C, Jimenez-Santos MJ, Garcia-Martin S, Carretero-Puche C, Garcia-Jimeno L, Ivanchuk V, Di Domenico T, Gomez-Lopez G, Al-Shahrour F (2021). Beyondcell: targeting cancer therapeutic heterogeneity in single-cell RNA-seq data. Genome Med.

[79] Tang Z, Liu X, Li Z, Zhang T, Yang B, Su J, Song Q. SpaRx: elucidate single-cell spatial heterogeneity of drug responses for personalized treatment. Brief Bioinform 2023, 24. 10.1093/bib/bbad338 PMC10555713 37798249

[80] Liu H, Gao J, Feng M, Cheng J, Tang Y, Cao Q, Zhao Z, Meng Z, Zhang J, Zhang G et al. Integrative molecular and spatial analysis reveals evolutionary dynamics and tumor-immune interplay of in situ and invasive acral melanoma. Cancer Cell 2024. 10.1016/j.ccell.2024.04.012 38759655

[81] Greenwald AC, Darnell NG, Hoefflin R, Simkin D, Mount CW, Gonzalez Castro LN, Harnik Y, Dumont S, Hirsch D, Nomura M (2024). Integrative spatial analysis reveals a multi-layered organization of glioblastoma. Cell.

[82] Hu J, Wang SG, Hou Y, Chen Z, Liu L, Li R, Li N, Zhou L, Yang Y, Wang L (2024). Multi-omic profiling of clear cell renal cell carcinoma identifies metabolic reprogramming associated with disease progression. Nat Genet.

[83] Cheng A, Xu Q, Li B, Zhang L, Wang H, Liu C, Han Z, Feng Z (2024). The enhanced energy metabolism in the tumor margin mediated by RRAD promotes the progression of oral squamous cell carcinoma. Cell Death Dis.

[84] Wang Y, Chen D, Liu Y, Shi D, Duan C, Li J, Shi X, Zhang Y, Yu Z, Sun N (2023). Multidirectional characterization of cellular composition and spatial architecture in human multiple primary lung cancers. Cell Death Dis.

[85] Xie L, Kong H, Yu J, Sun M, Lu S, Zhang Y, Hu J, Du F, Lian Q, Xin H (2024). Spatial transcriptomics reveals heterogeneity of histological subtypes between lepidic and acinar lung adenocarcinoma. Clin Transl Med.

[86] Szeitz B, Glasz T, Herold Z, Toth G, Balbisi M, Fillinger J, Horvath S, Mohacsi R, Kwon HJ, Moldvay J et al. Spatially resolved proteomic and transcriptomic profiling of anaplastic lymphoma kinase-rearranged Pulmonary Adenocarcinomas reveals Key players in Inter- and Intratumoral Heterogeneity. Int J Mol Sci 2023, 24. 10.3390/ijms241411369 PMC10380216 37511126

[87] Wu F, Zhang X, Wang M, Zhang J, Chen M, Ren Z, Wu M, Song P, Yu J, Chen D (2023). Deciphering the role of immunoglobulin secreting malignant lineages in the invasive frontiers of small cell lung cancer by scRNA-seq and spatial transcriptomics analysis. Cell Discov.

[88] Yang L, Zhang Z, Dong J, Zhang Y, Yang Z, Guo Y, Sun X, Li J, Xing P, Ying J, Zhou M (2023). Multi-dimensional characterization of immunological profiles in small cell lung cancer uncovers clinically relevant immune subtypes with distinct prognoses and therapeutic vulnerabilities. Pharmacol Res.

[89] Larroquette M, Guegan JP, Besse B, Cousin S, Brunet M, Le Moulec S, Le Loarer F, Rey C, Soria JC, Barlesi F et al. Spatial transcriptomics of macrophage infiltration in non-small cell lung cancer reveals determinants of sensitivity and resistance to anti-PD1/PD-L1 antibodies. J Immunother Cancer 2022, 10. 10.1136/jitc-2021-003890 PMC9125754 35618288

[90] Monkman J, Kim H, Mayer A, Mehdi A, Matigian N, Cumberbatch M, Bhagat M, Ladwa R, Mueller SN, Adams MN (2023). Multi-omic and spatial dissection of immunotherapy response groups in non-small cell lung cancer. Immunology.

[91] Song X, Xiong A, Wu F, Li X, Wang J, Jiang T, Chen P, Zhang X, Zhao Z, Liu H et al. Spatial multi-omics revealed the impact of tumor ecosystem heterogeneity on immunotherapy efficacy in patients with advanced non-small cell lung cancer treated with bispecific antibody. J Immunother Cancer 2023, 11. 10.1136/jitc-2022-006234 PMC9980352 36854570

[92] Ozato Y, Kojima Y, Kobayashi Y, Hisamatsu Y, Toshima T, Yonemura Y, Masuda T, Kagawa K, Goto Y, Utou M (2023). Spatial and single-cell transcriptomics decipher the cellular environment containing HLA-G + cancer cells and SPP1 + macrophages in colorectal cancer. Cell Rep.

[93] Qi J, Sun H, Zhang Y, Wang Z, Xun Z, Li Z, Ding X, Bao R, Hong L, Jia W (2022). Single-cell and spatial analysis reveal interaction of FAP(+) fibroblasts and SPP1(+) macrophages in colorectal cancer. Nat Commun.

[94] Wood CS, Pennel KAF, Leslie H, Legrini A, Cameron AJ, Melissourgou-Syka L, Quinn JA, van Wyk HC, Hay J, Roseweir AK (2023). Spatially resolved transcriptomics deconvolutes prognostic histological subgroups in patients with Colorectal Cancer and Synchronous Liver metastases. Cancer Res.

[95] Roelands J, van der Ploeg M, Ijsselsteijn ME, Dang H, Boonstra JJ, Hardwick JCH, Hawinkels L, Morreau H, de Miranda N (2023). Transcriptomic and immunophenotypic profiling reveals molecular and immunological hallmarks of colorectal cancer tumourigenesis. Gut.

[96] Pelka K, Hofree M, Chen JH, Sarkizova S, Pirl JD, Jorgji V, Bejnood A, Dionne D, Ge WH, Xu KH (2021). Spatially organized multicellular immune hubs in human colorectal cancer. Cell.

[97] Peng Z, Ye M, Ding H, Feng Z, Hu K (2022). Spatial transcriptomics atlas reveals the crosstalk between cancer-associated fibroblasts and tumor microenvironment components in colorectal cancer. J Transl Med.

[98] Wang F, Long J, Li L, Wu ZX, Da TT, Wang XQ, Huang C, Jiang YH, Yao XQ, Ma HQ (2023). Single-cell and spatial transcriptome analysis reveals the cellular heterogeneity of liver metastatic colorectal cancer. Sci Adv.

[99] Wu Y, Yang S, Ma J, Chen Z, Song G, Rao D, Cheng Y, Huang S, Liu Y, Jiang S (2022). Spatiotemporal Immune Landscape of Colorectal Cancer Liver Metastasis at single-cell level. Cancer Discov.

[100] Li R, Liu X, Huang X, Zhang D, Chen Z, Zhang J, Bai R, Zhang S, Zhao H, Xu Z (2024). Single-cell transcriptomic analysis deciphers heterogenous cancer stem-like cells in colorectal cancer and their organ-specific metastasis. Gut.

[101] Losic B, Craig AJ, Villacorta-Martin C, Martins-Filho SN, Akers N, Chen X, Ahsen ME, von Felden J, Labgaa I, D’Avola D (2020). Intratumoral heterogeneity and clonal evolution in liver cancer. Nat Commun.

[102] Zhou PY, Zhou C, Gan W, Tang Z, Sun BY, Huang JL, Liu G, Liu WR, Tian MX, Jiang XF (2023). Single-cell and spatial architecture of primary liver cancer. Commun Biol.

[103] Calderaro J, Ghaffari Laleh N, Zeng Q, Maille P, Favre L, Pujals A, Klein C, Bazille C, Heij LR, Uguen A (2023). Deep learning-based phenotyping reclassifies combined hepatocellular-cholangiocarcinoma. Nat Commun.

[104] Gan X, Dong W, You W, Ding D, Yang Y, Sun D, Li W, Ding W, Liang Y, Yang F (2024). Spatial multimodal analysis revealed tertiary lymphoid structures as a risk stratification indicator in combined hepatocellular-cholangiocarcinoma. Cancer Lett.

[105] Greten TF, Wang XW, Korangy F (2015). Current concepts of immune based treatments for patients with HCC: from basic science to novel treatment approaches. Gut.

[106] Wu R, Guo W, Qiu X, Wang S, Sui C, Lian Q, Wu J, Shan Y, Yang Z, Yang S (2021). Comprehensive analysis of spatial architecture in primary liver cancer. Sci Adv.

[107] Liu Y, Xun Z, Ma K, Liang S, Li X, Zhou S, Sun L, Liu Y, Du Y, Guo X (2023). Identification of a tumour immune barrier in the HCC microenvironment that determines the efficacy of immunotherapy. J Hepatol.

[108] Wang YF, Yuan SX, Jiang H, Li ZX, Yin HZ, Tan J, Dai ZH, Ge CM, Sun SH, Yang F (2022). Spatial maps of hepatocellular carcinoma transcriptomes reveal spatial expression patterns in tumor immune microenvironment. Theranostics.

[109] Wu L, Yan J, Bai Y, Chen F, Zou X, Xu J, Huang A, Hou L, Zhong Y, Jing Z (2023). An invasive zone in human liver cancer identified by stereo-seq promotes hepatocyte-tumor cell crosstalk, local immunosuppression and tumor progression. Cell Res.

[110] Chew V (2023). Unveiling the hidden battlefield: dissecting the invasive zone in liver cancer. Cell Res.

[111] Sun Y, Wu P, Zhang Z, Wang Z, Zhou K, Song M, Ji Y, Zang F, Lou L, Rao K (2024). Integrated multi-omics profiling to dissect the spatiotemporal evolution of metastatic hepatocellular carcinoma. Cancer Cell.

[112] Siegel RL, Miller KD, Wagle NS, Jemal A (2023). Cancer statistics, 2023. CA Cancer J Clin.

[113] Harbeck N, Gnant M (2017). Breast cancer. Lancet.

[114] Allred DC (2010). Ductal carcinoma in situ: terminology, classification, and natural history. J Natl Cancer Inst Monogr.

[115] Casasent AK, Schalck A, Gao R, Sei E, Long A, Pangburn W, Casasent T, Meric-Bernstam F, Edgerton ME, Navin NE (2018). Multiclonal Invasion in breast tumors identified by Topographic single cell sequencing. Cell.

[116] Nagasawa S, Kuze Y, Maeda I, Kojima Y, Motoyoshi A, Onishi T, Iwatani T, Yokoe T, Koike J, Chosokabe M (2021). Genomic profiling reveals heterogeneous populations of ductal carcinoma in situ of the breast. Commun Biol.

[117] Dent R, Trudeau M, Pritchard KI, Hanna WM, Kahn HK, Sawka CA, Lickley LA, Rawlinson E, Sun P, Narod SA (2007). Triple-negative breast cancer: clinical features and patterns of recurrence. Clin Cancer Res.

[118] Bassiouni R, Idowu MO, Gibbs LD, Robila V, Grizzard PJ, Webb MG, Song J, Noriega A, Craig DW, Carpten JD (2023). Spatial transcriptomic analysis of a diverse patient cohort reveals a conserved Architecture in Triple-negative breast Cancer. Cancer Res.

[119] Donati B, Reggiani F, Torricelli F, Santandrea G, Rossi T, Bisagni A, Gasparini E, Neri A, Cortesi L, Ferrari G (2024). Spatial distribution of Immune cells drives resistance to Neoadjuvant Chemotherapy in Triple-negative breast Cancer. Cancer Immunol Res.

[120] Tashireva LA, Kalinchuk AY, Gerashchenko TS, Menyailo M, Khozyainova A, Denisov EV, Perelmuter VM. Spatial Profile of Tumor Microenvironment in PD-L1-Negative and PD-L1-Positive triple-negative breast Cancer. Int J Mol Sci 2023, 24. 10.3390/ijms24021433 PMC9862087 36674951

[121] Kang K, Wu Y, Han C, Wang L, Wang Z, Zhao A (2023). Homologous recombination deficiency in triple-negative breast cancer: multi-scale transcriptomics reveals distinct tumor microenvironments and limitations in predicting immunotherapy response. Comput Biol Med.

[122] Kolbeinsson HM, Chandana S, Wright GP, Chung M (2023). Pancreatic Cancer: a review of current treatment and Novel therapies. J Invest Surg.

[123] Moncada R, Barkley D, Wagner F, Chiodin M, Devlin JC, Baron M, Hajdu CH, Simeone DM, Yanai I (2020). Integrating microarray-based spatial transcriptomics and single-cell RNA-seq reveals tissue architecture in pancreatic ductal adenocarcinomas. Nat Biotechnol.

[124] Cui Zhou D, Jayasinghe RG, Chen S, Herndon JM, Iglesia MD, Navale P, Wendl MC, Caravan W, Sato K, Storrs E (2022). Spatially restricted drivers and transitional cell populations cooperate with the microenvironment in untreated and chemo-resistant pancreatic cancer. Nat Genet.

[125] Yousuf S, Qiu M, von Voith L, Hulkkonen J, Macinkovic I, Schulz AR, Hartmann D, Mueller F, Mijatovic M, Ibberson D (2023). Spatially resolved Multi-omics single-cell analyses inform mechanisms of Immune Dysfunction in Pancreatic Cancer. Gastroenterology.

[126] Croft W, Pearce H, Margielewska-Davies S, Lim L, Nicol SM, Zayou F, Blakeway D, Marcon F, Powell-Brett S, Mahon B et al. Spatial determination and prognostic impact of the fibroblast transcriptome in pancreatic ductal adenocarcinoma. Elife 2023, 12. 10.7554/eLife.86125 PMC10361717 37350578

[127] Liebl F, Demir IE, Mayer K, Schuster T, D’Haese JG, Becker K, Langer R, Bergmann F, Wang K, Rosenberg R (2014). The impact of neural invasion severity in gastrointestinal malignancies: a clinicopathological study. Ann Surg.

[128] Xue M, Zhu Y, Jiang Y, Han L, Shi M, Su R, Wang L, Xiong C, Wang C, Wang T (2023). Schwann cells regulate tumor cells and cancer-associated fibroblasts in the pancreatic ductal adenocarcinoma microenvironment. Nat Commun.

[129] Weitz J, Garg B, Martsinkovskiy A, Patel S, Tiriac H, Lowy AM (2023). Pancreatic ductal adenocarcinoma induces neural injury that promotes a transcriptomic and functional repair signature by peripheral neuroglia. Oncogene.

[130] Karamitopoulou E, Wenning AS, Acharjee A, Zlobec I, Aeschbacher P, Perren A, Gloor B (2023). Spatially restricted tumour-associated and host-associated immune drivers correlate with the recurrence sites of pancreatic cancer. Gut.

[131] Andrieux G, Das T, Griffin M, Straehle J, Paine SML, Beck J, Boerries M, Heiland DH, Smith SJ, Rahman R, Chakraborty S (2023). Spatially resolved transcriptomic profiles reveal unique defining molecular features of infiltrative 5ALA-metabolizing cells associated with glioblastoma recurrence. Genome Med.

[132] Wang L, Jung J, Babikir H, Shamardani K, Jain S, Feng X, Gupta N, Rosi S, Chang S, Raleigh D (2022). A single-cell atlas of glioblastoma evolution under therapy reveals cell-intrinsic and cell-extrinsic therapeutic targets. Nat Cancer.

[133] Loussouarn D, Oliver L, Salaud C, Samarut E, Bourgade R, Beroud C, Morenton E, Heymann D, Vallette FM. Spatial distribution of Immune cells in primary and recurrent glioblastoma: a small case study. Cancers (Basel) 2023, 15. 10.3390/cancers15123256 PMC10297152 37370866

[134] Goswami S, Raychaudhuri D, Singh P, Natarajan SM, Chen Y, Poon C, Hennessey M, Tannir AJ, Zhang J, Anandhan S (2023). Myeloid-specific KDM6B inhibition sensitizes glioblastoma to PD1 blockade. Nat Cancer.

[135] Mei Y, Wang X, Zhang J, Liu D, He J, Huang C, Liao J, Wang Y, Feng Y, Li H (2023). Siglec-9 acts as an immune-checkpoint molecule on macrophages in glioblastoma, restricting T-cell priming and immunotherapy response. Nat Cancer.

[136] Ravi VM, Neidert N, Will P, Joseph K, Maier JP, Kuckelhaus J, Vollmer L, Goeldner JM, Behringer SP, Scherer F (2022). T-cell dysfunction in the glioblastoma microenvironment is mediated by myeloid cells releasing interleukin-10. Nat Commun.

